# Jahn–Teller Distortion Induced Mn^2+^‐Rich Cathode Enables Optimal Flexible Aqueous High‐Voltage Zn‐Mn Batteries

**DOI:** 10.1002/advs.202004995

**Published:** 2021-05-05

**Authors:** Lixin Dai, Yan Wang, Lu Sun, Yi Ding, Yuanqing Yao, Lide Yao, Nicholas E. Drewett, Wei Zhang, Jun Tang, Weitao Zheng

**Affiliations:** ^1^ Department of Polymer Science College of Chemistry Electron Microscopy Center Jilin University Changchun 130012 China; ^2^ School of Materials Science & Engineering Key Laboratory of Mobile Materials MOE Jilin University Changchun 130012 China; ^3^ NanoSpin Department of Applied Physics Aalto University Aalto FI‐00076 Finland; ^4^ CIC energiGUNE Albert Einstein 48 Vitoria‐Gasteiz 01510 Spain

**Keywords:** Jahn–Teller distortion, Mn2+‐rich cathodes, reversible Mn^2+^/Mn^4+^, Zn batteries, 2‐pH hydrogel electrolytes

## Abstract

Although one of the most promising aqueous batteries, all Zn‐Mn systems suffer from Zn dendrites and the low‐capacity Mn^4+^/Mn^3+^ process (readily leading to the occurrence of Jahn–Teller distortion, which in turn causes structural collapse and voltage/capacity fading). Here, the Mn^3+^ reconstruction and disproportionation are exploited to prepare the stable, Mn^2+^‐rich manganese oxides on carbon‐cloth (CMOs) in a discharged state through an inverted design, which promotes reversible Mn^2+^/Mn^4+^ kinetics and mitigates oxygen‐related redox activity. Such a 1.65 V Mn^2+^‐rich cathode enable constructing a 2.2 V Zn‐Mn battery, providing a high area capacity of 4.16 mA h cm^–2^ (25 mA h cm^–2^ for 10 mL electrolyte) and superior 4000‐cycle stability. Moreover, a flexible hybrid 2.7 V Zn‐Mn battery is constructed using 2‐pH hydrogel electrolytes to demonstrate excellent practicality and stability. A further insight has been gained to the commercial application of aqueous energy storage devices toward low‐cost, high safety, and excellent energy density.

## Graphical abstract

The Mn^2+^‐rich cathode is in discharge state caused by Jahn–Teller distortion, which promotes reversible Mn^2+^/Mn^4+^ kinetics and mitigates oxygen‐related redox, exhibiting wide electrochemical window, high cycle stability, and high area capacity independent of strong acids. 2‐pH hydrogel electrolytes are made to effectively improve the stability of flexible 2.7 V hybrid Zn batteries.

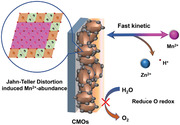

## 1 Introduction

Aqueous energy storage systems that exploit resource‐rich ions (e.g., Na^+^, K^+^, Zn^2+^, Mg^2+^, Ca^2+^, Al^3+^) have received widespread attention due to the resource cost and limited security of lithium batteries.^[^ ^1^ ^]^ Indeed, Zn‐manganese dioxide (Zn‐MnO_2_) batteries have been considered as an attractive alternative because of the high abundance of constituents, intrinsic safety, low cost, and a high theoretical capacity (308 mAh g^–1^).^[^ ^2^ ^]^ It is generally accepted that a reversible insertion/extraction of Zn^2+^ and/or H^+^ into MnO_2_ occurs during cycling, even in neutral electrolytes.^[^ ^3^ ^]^ For a conventional Zn‐Mn battery, the Mn cathode typically exists in a charged state (i.e., high valence Mn in MnO_2_). The low‐capacity Mn^4+^/Mn^3+^ process, however, requires a high redox, which results in oxygen defects and poor reaction kinetics. Indeed, the presence of Mn^3+^ leads to Jahn–Teller and its subsequent simultaneous disproportionation (2Mn^3+^ = Mn^2+^+ Mn^4+^),^[^ ^4^ ^]^ resulting in voltage and capacity fade accompanied by asymmetric changes in the Mn‐O bond lengths of [MnO_6_] octahedra in the [MnO_2_] layers and dissolution of Mn^2+^ into the electrolyte.^[^ ^5^ ^]^

Recently, considerable effort has been made to introduce the reversible Mn^4+^/Mn^2+^ couple into the Zn‐Mn batteries to obtain higher capacity and stability.^[^ ^6^ ^]^ However, the reaction Mn^2+^ + 2H_2_O ↔ MnO_2_ + 4H^+^ + 2e^–^ (1.228 V vs. SHE) blocks the high potential basis for Zn‐Mn batteries; as a result, it may cause the electrode to suffer from oxygen‐related side reactions at high voltages. Consequently, it is critical to reduce the valence of oxides, even leaving the electrodes in a discharged state.^[^ ^7^ ^]^ Thus, it is of great interest to obtain the reduction to Mn^2+^ by the utilization of discharge products, or by introducing high‐valence cations (e.g., Nb^5+^ and Ti^4+^) which are expected to reduce oxygen‐correlated redox and obtain higher voltage and capacity.^[^ ^7^, ^8^ ^]^ But such approaches lead to equilibration issues during deposition/dissolution of manganese oxides, due to the irreversible dissolution of Mn^2+^.^[^ ^6^, ^7^ ^]^ Moreover, it is also necessary to increase the concentration of Mn^2+^ in the electrolyte to keep the electrode in a discharged state while inhibiting Zn dendrites, as the interplay of electrode and electrolyte plays a significant role in the cycle stability of batteries.^[^ ^6^, ^9^ ^]^ However, this will also inevitably lead to water contact with the current collector. Thus, this cannot effectively suppress oxygen‐related side reactions, causing the irreversibility of the active materials beyond high‐voltage cycling in a Zn‐Mn battery.^[^ ^6b^ ^]^

Another crucial parameter is that the strongly acidic (pH = 1) nature will bring inherent risks, such as the corrosion of zinc anode and the occurrence of hydrogen evolution reaction (HER). One common solution to this problem involves decoupling electrolytes to enhance the stability of cathode and anode.^[^ ^10^ ^]^ This demonstrates that should the oxygen evolution reaction (OER) and HER be effectively suppressed, a large electrochemical window may be obtained. However, such complicated processes and their expensive costs may be necessarily prohibitive to pushing the high‐voltage battery into large‐scale application.

Herein, a direct‐architected multivalent manganese oxide is prepared by using electrodeposition and subsequently used as a novel 1.65 V Mn^2+^‐rich cathode for Zn battery. It is critical to introduce Mn^2+^ in the manganese oxides@carbon cloth (CMOs) via reversing the unfavorable Jahn–Teller effect (the Mn^3+^ reconstruction/disproportionation accompanied by the Mn^2+^ migration), i.e., the cathode in a discharge state. Our density functional theory (DFT) calculations, ex‐situ X‐ray photoelectron spectroscopy (XPS), and electron energy loss spectrum (EELS) reveal that the abundant Mn^2+^ in the electrode effectively promotes the reversible Mn^4+^/Mn^2+^ redox kinetics, H^+^/Zn^2+^ insertion/extraction kinetics, and reduces oxygen‐related redox. Integrated with 2 m ZnSO_4_ + 0.5 m MnSO_4_ electrolyte, our stable Zn‐Mn battery achieves a high area capacity of 4.16 mAh cm^–2^ (25 mA h cm^–2^ for 10 mL electrolyte) and an excellent cycle life (up to 4000 cycles with 85.1% capacity retention). Furthermore, we demonstrate a flexible 2.2 V solid‐state battery (FSZB) with a single hydrogel electrolyte (electrochemical stability window (ESW) of 2.65 V) and 2.7 V hybrid battery (FHZB) with a 2‐pH hydrogel electrolyte (ESW of 3.46 V), both of which maintain high area capacity, rate performance, and stability. Our intention is to motivate a new universal approach to developing practical aqueous batteries aimed toward safer, large‐scale energy storage applications.

## 2 Results and Discussion

### 2.1 Structural Characterization of CMOs

**Figure 1:**
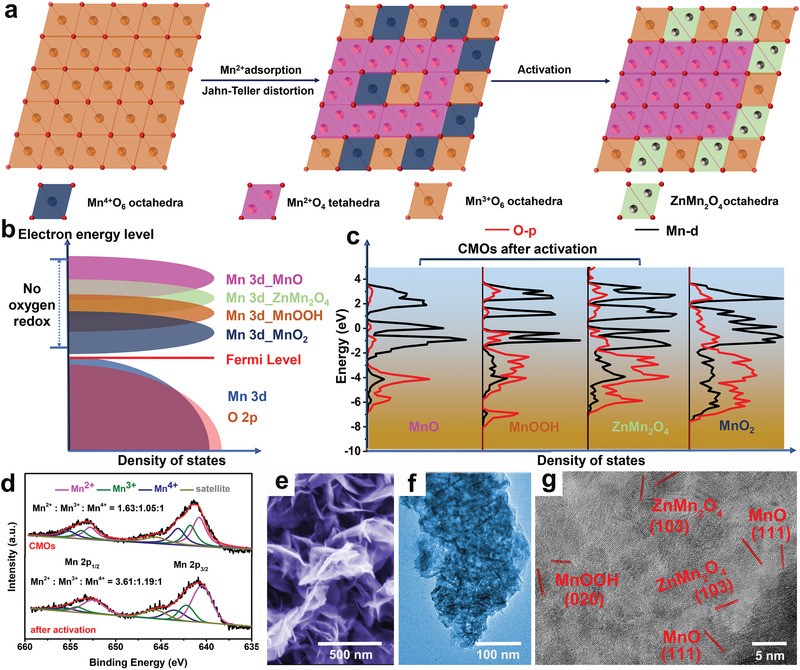
Design and structural characterization of CMOs. a) Preparation process and simulated structure of CMOs; b) schematic band structure of activation product and MnO_2_; c) partial density of states (PDOS) of the O‐*p* band (red) and Mn‐*d* band (black) of MnO, MnOOH, ZnMn_2_O_4_, and MnO_2_; d) X‐ray photoelectron spectroscopy (XPS) of Mn 2p; e) scanning electron microscopy (SEM) image; f) and g) high‐resolution transmission electron microscopy (HRTEM) image of CMOs after activation.

The initial CMOs are deposited on a clean carbon cloth (CC) by a typical electrodeposition method with 0.2 M MnSO_4_ electrolyte. In commonly used deposition methods, Mn^2+^ is directly oxidized to Mn(OH)_3_ and MnO_2_. In the process of Mn(OH)_3_ disproportionation, oxygen vacancies are generated and Mn^2+^ migrates (resulting in a [MnO_4_] tetrahedron in coordination with the 4 oxygen atoms) and rearranges via sliding the [MnO_2_] layer to form a stable spinel structure (**Figure** **1a**).^[^ ^6^, ^7^, ^11^ ^]^ Subsequently, CMOs were activated via 10‐cycle cyclic voltammetry (CV), which converts the remaining MnO_2_ into stable MnOOH and ZnMn_2_O_4_, leaving the electrode in the discharge state. This process effectively protects the Mn^2+^ inside CMOs, and the additional activation products increase the average valence of the electrode (Figure 1a), as high practical capacity requires a higher average valence of the transition metal.^[^ ^12^ ^]^

It was also found that the unique electrodeposition creates oriented CMOs. Furthermore, the presence of Mn^2+^ in the electrolyte effectively inhibits the dissolution of Mn^2+^ from the electrode. X‐ray diffraction (XRD) confirmed the successful synthesis of the disordered multivalent composition, MnO_2_ (JCPDS #44‐0141), with the typical (100) and (101) peaks at 37.18° and 42.48° (Figure S1, Supporting Information). Due to 3D structure of the CC substrate, the formed compound is present as the disordered rock salt phases, chemically similar to the target component. The stability and electrode reaction kinetics of CMOs were improved by 10‐cycle CV activation (−0.4 to 1.5 V). After activation, many small peaks appeared in the XRD pattern of CMOs which were attributed to MnOOH and ZnMn_2_O_4_ (JCPDS #74‐1049, #24‐1133) formation due to H^+^/Zn^2+^ embedded MnO_2_ on the surface (Figure S1, Supporting Information). Importantly, the MnO peaks appear with the typical (111) peaks at 35.24° (JCPDS #75‐0257).

DFT calculations were carried out to analyze the effect of Jahn–Teller distortion in activation process. A high‐capacity battery requires a high‐valence band, and the valence bands of MnOOH, ZnMn_2_O_4_, and MnO increased sequentially after activation, as shown in Figure 1b. Inside the CMOs, the O vacancies caused by the stacking of low‐valence activation products can effectively reduce the local oxygen mobility triggered by O redox, thereby ensuring less water redox during the long‐term cycling.^[^ ^13^ ^]^ In addition, partial density of states (PDOS) analysis (Figure 1c and S2, Supporting Information) shows that the O‐p bands of MnO and MnOOH is closer to the Fermi level than those in MnO_2_. As a result, it facilitates a more active electron transfer dynamics.^[^ ^6b^ ^]^

XPS was carried out on the CMOs, and the Mn 2p spectrum was analyzed with respect to the ratio of each valence state (Figure 1d). High‐resolution XPS of Mn 2p was deconvoluted into two parts: Mn 2P_1/2_ and Mn 2P_3/2_. The coupled peaks of Mn 2P_3/2_ (Mn^2+^, Mn^3+^, Mn^4+^) appear at 640.5, 642.4, and 643.7 eV, while the coupled peaks of Mn 2P_1/2_ are split at 655.4, 654.1, and 652.1 eV, respectively. The remaining peak at 645.6 eV is associated with the satellite peak. In CMOs, the calculated ratio is Mn^2+^ : Mn^3+^ : Mn^4+^ = 1.63:1.05:1, which shows that a large amount of Mn^2+^ exists in the cathode. After activation, the calculated ratio is Mn^2+^ : Mn^3+^ : Mn^4+^ = 3.61:1.19:1, illustrating that the average valence state of the electrode is lower and closer to the discharge state. During the activation process, the reversible Mn^2+^/Mn^4+^ and insertion/extraction reaction will continuously optimize the electrode structure and improve the electrode reaction kinetics. To verify this positive impact, we performed ultraviolet photoelectron spectroscopy (UPS) tests on both the CMOs and the activated CMOs (Figure S3, Supporting Information). After activation, a blue shift from 4.79 to 2.38 eV was observed in the onset region (*E*_onset_), while there was almost no change in the cutoff region (*E*_cutoff_). Using the formula *ϕ* = hn − (*E*_Cutoff _ − *E*_onset_), it can be concluded that work function (*ϕ*) decreases from 8.0 to 5.59 eV, showing high electron transport ability, which is consistent with our DFT analysis.^[^ ^14^ ^]^ In combination with the above XPS and XRD results, the O vacancy in CMOs increase after activation, i.e., the electron activation energy decreases, promoting the electrical conductivity of the material.

SEM showed the materials adopt a homogeneous nanobelt morphology, and grew vertically on the CC (Figure 1e and S4a,b, Supporting Information). Transmission electron microscopy (TEM) images also confirm their anisotropy, uniform morphology, and high aspect ratio, which provide a basis for reversible insertion/extraction of ions (Figure 1e,f). The high‐resolution TEM (HRTEM) image in Figure S4c (Supporting Information) confirms the interplanar spacings of (211) and (310) crystal planes of MnO_2_, MnO (111), and MnOOH (410) for original CMOs, which are coincident with the XRD results. The interplanar spacings of MnO (111), MnOOH (020), and ZnMn_2_O_4_ (103) for CMOs after activation are identified in Figure 1g. Furthermore, Figure S5 (Supporting Information) shows a high‐angle annular dark‐field scanning transmission electron microscopy (HAADF‐STEM) image of the original CMOs and corresponding elemental maps, which implies the coexistence of elemental C, Mn, O, and Zn. All the above results indicate that CMOs is in discharge state.

### 2.2 Mechanism of CMOs Widening Electrochemical Window

**Figure 2:**
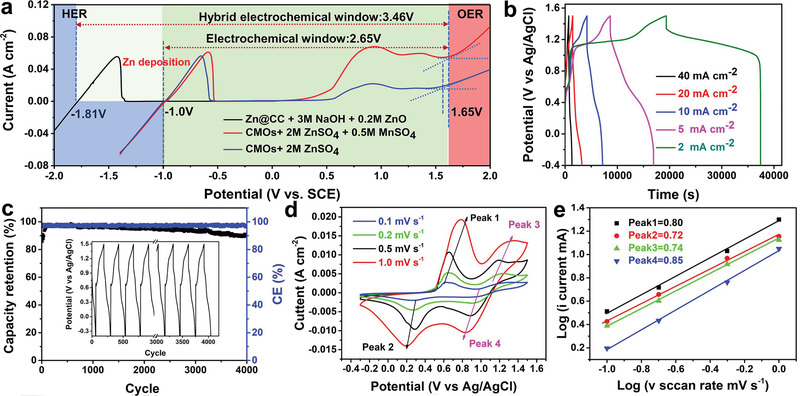
Performance assessment of the tri‐electrode Zn/CMOs cell. a) Verification of the electrochemical stability window to ensure successful operation. Hydrogen evolution reaction (HER) and oxygen evolution reaction (OER) properties were obtained from linear sweep voltammetry (LSV) analysis at a scan rate of 5 mV s^–1^; b) GCD profiles at 2–40 mA cm^–2^; c) long‐term cycling performances cycle performance at 10 mA cm^–2^ (the insert shows the voltage profiles at different cycles); d) cyclic voltammetry (CV) curves at 0.1–1.0 mV s^–1^; e) log(*i*) versus log(*v*) plots at specific peak currents.

Linear sweep voltammetry (LSV) was employed to further explore the Zn battery mechanisms which produce a high operating voltage of 2.2 V (Figure S6, Supporting Information). The reactions of parasitic H_2_ (Zn anode) and O_2_ (Mn cathode) were significantly suppressed to −1.0 and 1.35 V, respectively. When manganese sulfate was added to the electrolyte, the OER was suppressed to 1.51 V as the concentration of Mn^2+^ gradually increased to 0.5 m. It is worth noting that MnO_x_ deposition is separated from O_2_ precipitation by the addition of MnSO_4_. When Pt is replaced with CC, the OER can be increased to 1.61 V, providing the basis for high voltage Zn batteries. When the CMOs were investigated via LSV, the OER appeared after 1.65 V (**Figure** **2a**). When Mn^2+^ in solution forms MnO_2_ on the electrode surface, water molecules cannot directly contact CC, because the original multivalent manganese (+2 and +3) in the electrodes can be directly oxidized to tetravalent manganese. As a consequence, it reduces the possibility of O participating in the reaction – thus bypassing the oxygen‐related redox activity.^[^ ^12^, ^13^ ^]^ When the electrolyte of the negative electrode consists of 3 m NaOH and 0.2 m ZnO, HER is delayed to −1.81 V. Thus, an ESW of 3.46 V can be obtained. It should be noted that, in the long‐term charging and discharging process, O_2_ will inevitably participate in the reaction related to the solubility/deposition of manganese dioxide.^[^ ^15^ ^]^

To demonstrate the mechanism by which CMOs inhibit oxygen‐related redox reaction, a tri‐electrode cell was constructed which was comprised of a cathode (CMOs), an anode (Zn@CC), an Ag/AgCl (reference electrode), and a Mn^2+^/Zn^2+^ hybrid electrolyte (2 m ZnSO_4_ + *x* m MnSO_4_, *x* = 0.2, 0.5, 1 m, respectively) (shown in Figure S7a, Supporting Information). At a scan rate of 5 mV s^–1^, the CMOs exhibit two pairs of redox peaks at −0.3 to 1.5 V, as shown in Figure S7a (Supporting Information) (0.68/0.23 and 1.43/0.76 versus Ag/AgCl, respectively), which is attributed to the reversible 2‐electronic reaction (Mn^2+^/Mn^4+^ reaction) and Zn^2+^/H^+^insertion/extraction^[^ ^9^, ^11^ ^]^. When the electrolyte is only 2 m ZnSO_4_ the two pairs of redox peaks are still easily observed, indicating that both the reactions are still present, so a large amount of Mn^2+^ is stored in the electrode. But after 400 cycles at 10 mA cm^–2^ the capacity decreases to 86.8% due to the irresistible dissolution of Mn^2+^ (Figure S7b,c, Supporting Information). When the electrolyte was changed to 0.5 m MnSO_4_ the peak (after 1.2V in CV curves) may be attributed to OER (Figure S7d, Supporting Information). When the pH was adjusted to be equal to 2 m ZnSO_4_ + 0.5 m MnSO_4_, OER will be easily ignored, but the entire CV curve tends to be shaped like a shuttle and is irreversible, and OER is still found at 1.3 V.

A clean CC was used as a cathode to detect the presence of O in the reaction.^[^ ^6b^ ^]^ There was no oxygen‐related reaction with the CC during the charging process, demonstrated by XPS. As shown in Figure S8 (Supporting Information), during the charge/discharge process there is no appearance of C=O peak (287.2 eV); instead only C−C (284.8 eV), C−OH (286.1 eV), and C−Mn peaks (282.5 eV) are visible. In principle, after CC was coated with GO there should be a large amount of C=O on the CC surface. When it was used as the cathode for CV test, an obvious OER peak appeared after 1.2 V (Figure S9, Supporting Information). This suggests that the increased O content in the electrode may lead to OER production. In summary, the totality of the above available data strongly indicates that the oxygen‐related redox is effectively suppressed.

When the Mn^2+^ content in the solution gradually increases, the capacity concurrently increases. Notably, no obvious oxygen reduction reaction was detected during the activation process with 2 m ZnSO_4_ + 0.5 m MnSO_4_, and CMOs held the approximately highest value (1.5 V) for a Mn cathode in an aqueous Zn battery.^[^ ^2^, ^10^, ^16^ ^]^ However, when the concentration of Mn^2+^ in the solution reached 1 m, the OER (≈1.5V) appeared, and the potential widow faded to 1.2 V (Figure S7a, Supporting Information). Thus, it is reasonable to conclude the Zn‐Mn battery comprised of a 2 m ZnSO_4_ + 0.5 m MnSO_4_ electrolyte, Zn anode, and CMOs is controlled by a 2‐electron reaction and a classical Zn^2+^/H^+^ insertion/extraction process. The capacity increased to a constant value after 10 cycles of CV activation (Figure S10a, Supporting Information). In the galvanostatic charge/discharge tests, CMOs showed two distinct platforms between −0.3 and 1.5 V, a Coulomb efficiency (CE) of 96.8%, and a higher discharge capacity of 589.6 mAh g^–1^ at 2 mA cm^–2^ (Figure 2b). Notably, 93.2% capacity retention can be achieved at 10 mA cm^–2^ after 4000 cycles, and two redox peaks remained visible in the CV curves after long‐term cycle (Figure S10b, Supporting Information). When the tri‐electrode cell was charged for 3 h, 25 mAh cm^–2^ can be reached for CMOs as abundant Mn^2+^ in the 10 mL hybrid electrolyte continuously deposits on the electrode (Figure S10c, Supporting Information).^[^ ^6a^ ^]^ The layered CMOs enable an effective inhibition of the otherwise random distribution induced Jahn–Teller distortion, leading to a good contact between MnO_2_ and the conductive substrate. As a result, it is beneficial to the reversibility and kinetics of the Mn^2+^/Mn^4+^ reduction and insertion/extraction process.^[^ ^5c^ ^]^ In order to verify this point of view, an XPS test was performed after 4000 cycles of CMOs (Figure S10d,e, Supporting Information). Some Mn^2+^ will dissolve in the electrolyte, but there is abundant Mn^2+^ in CMOs, and the calculated ratio is Mn^2+^ : Mn^3+^ : Mn^4+^ = 0.44:1.15:1. In the Mn 3s spectrum, the spin‐energy splitting (Δ*E*) is 5.4 eV, indicating the main valence state of the CMO = 3 (MnO, MnOOH, and ZnMn_2_O_4_). It is important that the ratio of Mn^3+^ to Mn^4+^ is almost unchanged, which verifies that the distortion is effectively suppressed during the cycle life. This proves that our electrode reaction maintains excellent kinetics and stability during the long life span.

To further clarify the charge transfer dynamics, the CMOs were tested at different scan rates from 0.1 to 1 mV s^–1^ (Figure 2d). The relationship between peak current and scan rate can be attributed to: *i = av^b^* (converted to *log (i) = blog(v) + log(a)*), where a value of *b* near 0.5 indicates diffusion control and a value near 1 indicates surface control.^[^ ^15^ ^]^ When the scan rate is less than 1 mV s^–1^, the *b* value corresponds to 0.80, 0.72, 0.74, and 0.85. The higher *b* value proves that the capacity is mainly controlled by the capacitive charge storage (Mn^2+^/Mn^4+^, 2‐electron reaction) with good kinetics, and that the ion insertion/extraction plays an complementary role.^[^ ^4^, ^6^, ^10^ ^]^

### 2.3 Electrochemical Performance of Zn//CMOs Button Cell (ZCBC)

**Figure 3:**
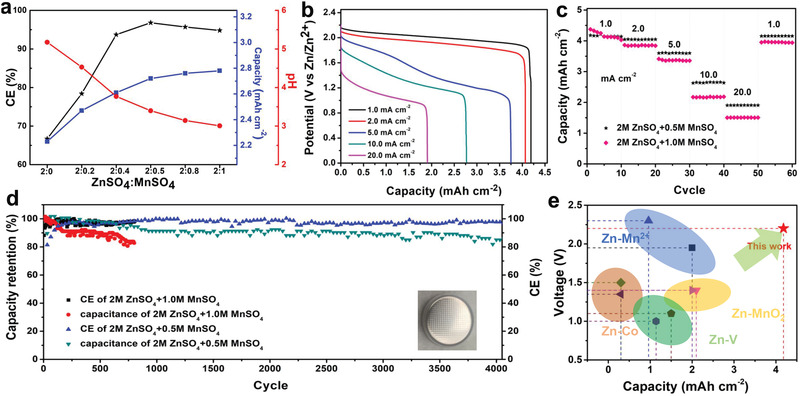
Electrochemical performances of the novel high‐voltage ZCBC. a) The relationship of Coulomb efficiency (CE), pH, capacity, and Zn^2+^/Mn^2+^ hybrid electrolyte; b) GCD profiles at 1–20 mA cm^–2^; c) rate performance; d) cyclability plot – the inset is the photo of ZCBC; e) the comparison on the voltage and specific capacity of different Zn batteries.

In order to verify the practical application of CMOs, the CR 2016 coin‐type cells were assembled using the 2 m ZnSO_4_ + x m MnSO_4_ electrolyte to facilitate electrochemical performance characterization of the resulting Zn//CMOs button cell (ZCBC). As the Mn^2+^ concentration in the electrolyte increases, the pH gradually decreases. This is conducive to the 2‐electron reaction, and thus the capacity of ZCBC can further increase (**Figure** **3a**).^[^ ^6a^ ^]^ However, when the Mn^2+^ concentration increases to 1 m (pH =2.98), the CE will decrease. Consequently, 2 m ZnSO_4_ + 0.5 m MnSO_4_ (pH = 3.20) was used as representing an optimal electrolyte. It is possible that the divalent manganese in the electrode reduces the dependence of the battery reaction on low pH and high concentration of Mn^2+^. All ZCBC CV curves taken at different scanning rates showed broad redox peaks, which were caused by the transition of multivalent Mn (Figure S11, Supporting Information). As the scan rate increased, the shape of the CV peak became wider and the area increased without a significant polarization. Such occurrence is fully consistent with the Mn^2+^/Mn^4+^ reduction/oxidation and insertion/extraction kinetics.^[^ ^1b^ ^]^ At different current densities of 1.0, 2.0, 5.0, 10.0, and 20.0 mA cm^–2^, the ZCBC exhibited capacities of 4.16, 4.08, 3.74, 2.77, and 1.89 mAh cm^–2^, respectively, i.e., excellent rate performance (Figure 3b, c). Notably, the Mn^2+^/Zn^2+^ hybrid electrolyte successfully increased the cyclability of ZCBC to 4000 cycles while maintaining 85.1% retention as shown in Figure 3d. A comprehensive comparison of Zn battery with respect to the specific area capacity and average discharge voltage is given in Figure 3e, and it can be seen that the ZCBC has obviously advantageous properties.^[^ ^1^, ^2^, ^4^, ^6^, ^9^, ^17^ ^]^

Importantly, our ZCBC exhibits a voltage fade (*V*_fade_) of 0.05 V at 2 mA cm^–1^, which is smaller than previously reported for ZCBCs, proving the superiority of electrodes including embedded Mn^2+^ (Figure S12, Supporting Information).^[^ ^2^, ^6^ ^]^ *V*_fade_ = *V*_OCV_ + *V*_P_, where *V*_OCV_ is the open circuit voltage controlled by thermodynamics (composition), and *V*_P_ is the overpotential controlled by dynamics (kinetic resistance). During the first few cycles the charge/discharge voltage of the full‐cell 2.2 V Zn‐Mn battery showed asymmetry, and the CE was only 85.0% (Figure 3d) – that is, the oxidation platform was not recovered during the discharge. With the progress of activation this asymmetry gradually disappeared, the CE gradually reached 98.6%, and reversibility and stability was achieved by the CMOs cathode. The resistance of original ZCBC was 7.01 Ω, and 2.26 Ω decreased after activation. Even after 4000 cycles, the resistance increased by only 1.07 Ω, indicating that CMOs pre‐doped with Mn^2+^ have obtained fast kinetics reaction and effectively reduce *V*_P_ (Figure S13, Supporting Information).

The reaction can be controlled by changing the content of Mn^2+^ to adjust pH in the electrolyte.^[^ ^6b^ ^]^ A small amount of H_2_SO_4_ was added to the electrolyte (2 m ZnSO_4_ + 0.2 m MnSO_4_, pH = 4.53) to adjust the pH to 3.52 (2 m ZnSO_4_ + 0.5 m MnSO_4_), and the ZCBC showed enhanced capacity (shown in Figure S14, Supporting Information) presenting a discharge curve almost the same as that of 2 m ZnSO_4_ + 0.5 m MnSO_4_. The enhancement of capacity mainly comes from the promotion of H^+^ for two‐electron reaction and the insertion of H^+^ in CMOs, therefore, successfully regulating the reaction of the electrodes to control the performance of the battery. At the same time, using a high‐concentration salt to regulate pH can bypass the corrosion of anode (as we will discuss later) and enhance the cycling performance of the battery.^[^ ^9^ ^]^ Consequently, the reaction kinetics and stability of Zn‐Mn batteries were enhanced by regulating salt concentration to reduce the dependence of low pH.^[^ ^9^ ^]^

The preparation of electrodes on CC is intended to endow the battery with multifunctionality, and the 3D structure of CC can inhibit the formation of Zn dendrites.^[^ ^14^ ^]^ The galvanostatic cycling performance of the zinc symmetrical battery with Zn@CC was tested at 0.5 mA cm^–2^ (Figure S15a,b, Supporting Information), and all symmetrical cells exhibited a small degree of polarization for the original cycles. A hard short occurred less than 200 h in 1 m ZnSO_4_ cells, in contrast to the 1 m ZnSO4 with x m MnSO_4_ cells which ran stably for 400 h. With the increase of Mn^2+^ concentration, the polarization decreased notably (from 100 to 50 mV). When *x* = 0.5 or 1, the polarization apparently stopped decreasing. Without MnSO_4_ addition, dendrites were gradually produced after multiple cycles, while the surface of that with 0.5 m MnSO_4_ was still smooth (Figure S15c,d, Supporting Information). Moreover, the XRD pattern of Zn@CC after 400 h cycles in 2 m ZnSO_4_ + 0.5 m MnSO_4_ shows no differences to the original Zn@CC (Figure S16, Supporting Information). Thus, a high concentration of heteroatoms can effectively inhibit the growth of Zn dendrites, Mn^2+^ does the same thing here and the 3D structure of CC provides a place for reversible deposition of Zn and effectively enhances the stability.^[^ ^17c^ ^]^

### 2.4 Charge Storage Mechanism of ZCBC

**Figure 4:**
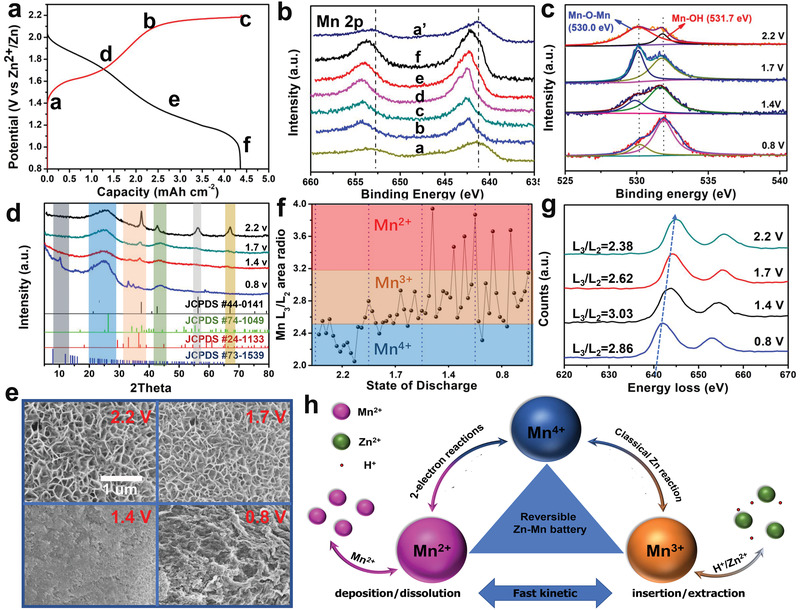
Simultaneous mechanism for ZCBC. a) Second charge/discharge profile for ZCBC; ex situ b) XPS Mn 2p, c) XPS O 1s, d) XRD, e) SEM; f) ratio of Mn L_3_/L_2_ peak area during the discharge process indicating the valence change of Mn ions; g) Mn‐L_2,3_ electron energy loss spectrum (EELS) spectra for average ratio of Mn L_3_/L_2_ peak area during the discharge process; h) simulating the change of valence state and mechanism during charging/discharging process.

To further explore the mechanism of CMOs, ex‐situ XPS (**Figure** **4a**) was used to characterize the morphology and structural evolution of the cathode in Zn‐Mn cells. Using 1.5 V cathode and 0.7 V Zn anode, a complete CV curve was measured in the range of 0.8−2.2 V (with the following data being recorded in that region). The oxidation state of Mn during discharge/charge was further verified by the Mn 2p spectrum of the manganese oxides (shown in Figure S7b, Supporting Information) under discharge (0.8 V) and charge (2.2 V) conditions. The Mn 2p and Zn 2p spectrum exhibited a reversible change of valence throughout the charge and discharge process (Figure 4b and S17a, Supporting Information). In the Mn 3s spectrum (Figure S17b, Supporting Information), Δ*E* of 4.7 eV suggests that the main valence state of Mn is 4 (MnO_2_) when charging to 2.2 V. Moreover, the Δ*E* values changed to 5.7 eV, indicating the main valence state of the CMO becomes 2 and 3. In the O 1s spectrum (Figure 4c), the two binding energy peaks correspond to the characteristic bands of the Mn‐O lattice and the C‐O on the CC, respectively. In addition, the O 1s energy spectrum can be fitted to the two components, which are related to the manganese‐hydrogen bond at Mn‐O absorbed at ≈531.7 eV and the manganese‐hydrogen bond at MnO_2_ at ≈529.9 eV. This implies that MnO_2_ on the surface is reduced to Mn^2+^, dissolved in the solution, and the Mn‐O bond is significantly stronger than other regions. It indicates an increase in Mn^3+^, as the result of H^+^ embedded in the cathode. A decrease in the Mn−O−Mn bond indicates that Mn^4+^ decreased, and Zn^2+^ could then be inserted in the region. The resulting insertion/extraction mechanism of H^+^ and Zn^2+^ at relatively low voltages is similar to that previously reported.^[^ ^3^, ^6^, ^17^ ^]^

Ex‐situ XRD and corresponding ex‐situ SEM (Figure 4d,e) show that MnO_2_ with different crystal structures was deposited. Consistent with the XPS results, MnOOH (JCPDS#74‐1049) and ZnMn_2_O_4_ (JCPDS #24‐1133) accordingly appeared during the discharge to 1.4 V. The formation of flake‐like ZnSO_4_[Zn(OH)_2_]_3_⋅5H_2_O (8–9°, 25–30°, and 32–38°) was accompanied by the reaction of MnO_2_ with protons in water to form MnOOH.^[^ ^1b^ ^]^ After H^+^ reacted with MnO_2_, the subsequent OH^–^ was then able to react with ZnSO_4_ and H_2_O in the aqueous electrolyte to form ZnSO_4_[Zn(OH)_2_]_3_⋅5H_2_O on the electrode. In brief, it reaches a neutral charge in the system.^[^ ^4b^ ^]^ For the ZCBC charged to 2.2V, HRTEM image in Figure S18a,b (Supporting Information) depicts the interplanar spacings of MnO_2_ (310) and MnOOH (020). When the ZCBC was discharging to 0.8 V, Figure S18c,d (Supporting Information) shows the interplanar spacings of MnO (111), MnOOH (410), and ZnMn_2_O_4_ (103) returned to the original state of CMOs after activation.

Hence, the electrochemical reactions of the aqueous Zn‐Mn batteries may be summarized as below:

Cathode:

1. 2‐electron reaction
  (1) $$\begin{array}{ccc} Mn^{2+} & + & 2H_{2}O\;\Leftrightarrow \;\mathrm{Mn}O_{2}+4H^{+}\\ & + & 2e^{-}\;\mathrm{formation}\;\mathrm{of}\;\mathrm{in}\;\mathrm{situ}\;\mathrm{Mn}O_{2} \end{array}$$
2. Classic Zn battery reaction
  (2) $$\mathrm{Mn}O_{2}\;+\;H^{+}\;+\;e^{-}\Leftrightarrow \mathrm{MnOOH}$$
  (3) $$2\mathrm{Mn}O_{2}+Zn^{2+}+2e^{-}\Leftrightarrow \mathrm{ZnM}n_{2}O_{4}$$
  (4) $$\begin{array}{ccc} 3Zn^{2+} & + & 6{\mathrm{OH}}^{-}\;+\;2\mathrm{ZnS}O_{4}\;+\;5H_{2}O\\ & \Leftrightarrow & 2\mathrm{ZnS}O_{4}{\left[\mathrm{Zn}{\left(\mathrm{OH}\right)}_{2}\right]}_{3}\;•\;5H_{2}O \end{array}$$

Anode:

(5) $$\mathrm{xZ}n^{2+}+2xe^{-}\Leftrightarrow \mathrm{xZn}$$

It should be noted that MnO peak was not sharp in XRD pattern, but Mn^2+^ was still detected in the XPS spectrum. This is likely due to Mn^2+^ on the surface having been oxidized during the sampling test. To further confirm the electrode reaction process (Figure 4f,g), ex‐situ Mn‐L_2,3_ EELS was used to explore the valence state changes during discharge from 2.2 to 0.8 V, as the intensity ratio of the excitation peaks of L3 (2p^3/2^‐3d) and L2 (2p^1/2^‐3d) in EELS is highly sensitive to transition metal ion valence changes. Since two reactions exist concurrently, we performed EELS tests on three samples with the same charge state and selected five positions for each sample for evaluation (Figure S19, Supporting Information). When charged to 2.2 V, the Mn (L_3_) average main peak gradually shifted from 644.9 to 644.0, 643.7, and 641.8 eV (Figure 4g), and the corresponding *L*_3_/*L*_2_ will gradually increase from 2.38 (Mn^4+^) to 2.62 (Mn^3+,4+^), 3.03 (Mn^2+,3+^), and 2.86 (Mn^2+,3+^) (Figure 4g).^[^ ^18^ ^]^ When discharged from 1.4 to 0.8 V, the increase of the Mn^3+^ ratio was due to the process of H^+^ and Zn^2+^ insertion into MnO_2_ after the dissolution of Mn^2+^.^[^ ^6c^ ^]^ This shows that the 2‐electron reaction followed with the classic Zn battery reaction (Figure 4h), which is consistent with the XPS, XRD, and SEM result. Pre‐positioning low‐valence Mn^2+^ into CMOs can enhance the electrode reaction kinetics (Mn^2+^/Mn^4+^ deposition/dissolution and H^+^/Zn^2+^ insertion/extraction) during the long‐term cycle (Figure 4h).

### 2.5 Flexible Solid‐State Zn‐Mn Battery and Hybrid Zn‐Mn Battery

**Figure 5:**
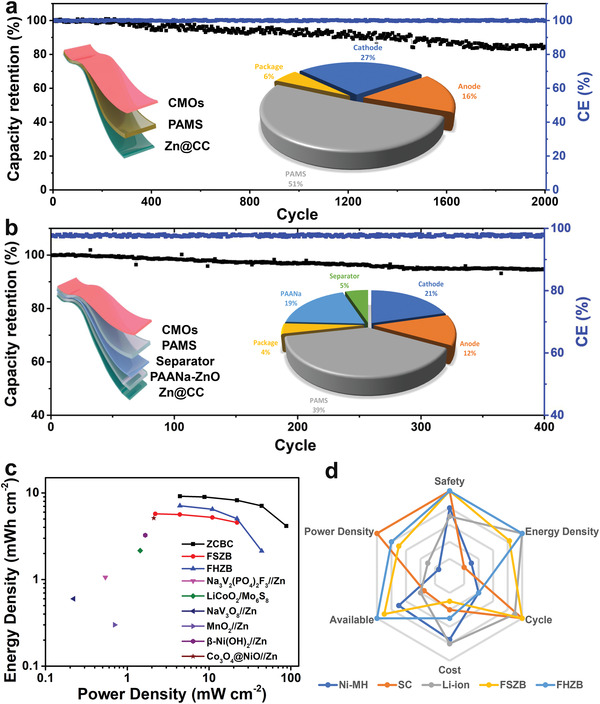
Electrochemical performance of flexible solid‐state Zn‐Mn battery (FSZB) and flexible hybrid Zn battery (FHZB). a) Cycle performance at 10 mA cm^–2^ for a) FSZB and b), the internal diagram shows the simulated FSZB & FHZB and the component proportion of FSZB & FHZB showing an ultrahigh loading of electrolyte in a whole device; c) Ragone plots showing the areal energy density and power density compared with reported results; d) A comprehensive comparison among supercapacitor (SC), Li‐ion battery (LiB), Ni‐MH battery, FSZB and FHZB.

Based on the excellent performance of the aqueous Zn‐Mn battery, it was deemed a promising candidate for use as a flexible solid‐state Zn‐Mn battery (FSZB) (**Figure** **5a**). FSZB can be flexibly assembled in an open‐air environment, facilitating large‐scale production. Specifically, the free‐standing deposited zinc on the CC is used as the anode, the free‐standing CMOs as the cathode, and the polyacrylamide‐cellulose (PAMS) hydrogel as the electrolyte.^[^ ^19^ ^]^ Here, a precise swelling method was applied to control the proportion of ions in the electrolyte – soaking 0.5 g dried PAMS hydrogel into 1 mL electrolyte (Zn:Mn = 2:0.5), as the PAMS gel can absorb up to a maximum 3 mL cm^–2^ of electrolyte. PAMS exhibits an excellent conductivity of 20.8 mS cm^–1^, which was evaluated by electrochemical impedance spectroscopy (EIS) (Figure S20a,b, Supporting Information).^[^ ^20^ ^]^ Although the storage and ion transport resistance of flexible electrolytes is less than Zn^2+^/Mn^2+^ solutions,^[^ ^21^ ^]^ the system still showed an excellent capacity (2.67 mAh cm^–2^ at 1.0 mA cm^–2^) superior to the majority of the reported literature based on the quality of Mn cathodes (Figure S21a, Supporting Information)^[^ ^2^, ^22^ ^]^. Even at 10 mA cm^–2^ FSZB maintains a capacity of 2.05 mAh cm^–2^, demonstrating a superior rate performance (Figure S21b, Supporting Information).^[^ ^20^ ^]^ This is due to the good ionic conductivity of the PAMS hydrogel electrolyte; that is, the highly porous network allows for rapid ion transport kinetics in charge and discharge process. At the same time, solid‐state Zn‐Mn battery exhibited a capacity retention of 84.6% after 2000 cycles (Figure 5a) under a large current density of 10 mA cm^–2^, demonstrating significant long‐term cycle stability. This remarkable long‐term cycle performance is mainly attributed to the excellent water retention of the PAM hydrogel electrolyte and interface contact between the electrode and the electrolyte.^[^ ^23^ ^]^ Moreover, the reliable toughness and flexibility of hydrogels provides results in devices well suited for practical applications (e.g., for use in safe, wearable electronic devices).^[^ ^24^ ^]^ The FSZB based on PAMS electrolyte exhibits outstanding flexibility and capacity retention when the battery was bent to 90° and 180° (Figure S21c, Supporting Information).

Using two different pH electrolytes effectively widens the electrochemical window and increases energy density, but also presents a challenge on account of the use of expensive and specialized separator membranes.^[^ ^10a,b^ ^]^ Here, we use the previously prepared PAMS electrolyte combined with sodium polyacrylate‐zinc oxide (PAANa‐ZnO) alkaline hydrogel electrolyte (Figure S20a,c, Supporting Information),^[^ ^25^ ^]^

using common cellophane as a separator, effectively solving the problem of neutralization of electrolyte caused by diaphragm ruptures (the anode reaction chemistry is Zn + 4OH^–^ ⇔ Zn(OH)_4_^2–^ + 2e^–^ E = −1.4 V vs. SHE). In Figure S21d,e (Supporting Information) and Figure 5b, the flexible hybrid Zn battery (FHZB) exhibits high operating voltage (2.7 V), high area capacity (2.63 mAh cm^–2^), superior rate performance and cyclability (86.7% capacity retention after 400 cycles). Similarly, good flexibility can be achieved for FHZB owing to the use of two flexible electrolytes (Figure S21f, Supporting Information). We suspect that the rate performance and cycling stability of FHZB could be further improved by optimizing the hydrogel structure to reconstruct the anolyte/catholyte relationship.

A Ragone plot was used to make a comprehensive summary on areal energy/power density, and our results excel among various reported aqueous batteries (Figure 5c and Table S1, Supporting Information). A simple soft‐pack battery was prepared for FSZB and FHZB, and the main material can account for 94% (FSZB) and 96% (FHZB), respectively. Both our FSZB and FHZB offer significant advantages in terms of packaging energy density, power density, cycling, availability, cost, and safety (Figure 5d).

## 3 Conclusion

Owing to the nanosheet morphology and the particularly appropriate interlayer spacing, the oriented Mn^2+^‐rich manganese oxide prepared through Jahn–Teller distortion effectively enhance Mn^2+^/Mn^4+^ electrolytic dynamics and H^+^/Zn^2+^ insertion, while also inhibiting OER in Zn‐Mn batteries, as demonstrated by DFT, ex‐situ XPS, XRD, and EELS. In addition, the replacement of high‐acid electrolytes with high‐concentration hybrid electrolyte ensures an excellent stability and compatibility of zinc anodes and CMOs cathode, leading to a high cycle stability. The assembled Zn‐Mn battery is capable of obtaining a voltage of 2.2 V, a high area capacity of 4.16 mAh cm^–2^, and long life‐span (85.1% capacity retention over 4000 cycles). We present the construction of a safe, flexible 2.7 V Zn‐Mn hybrid battery using 2‐pH hydrogel electrolytes, which has long‐term stability and superior rate performance. Our result highlights a pathway for the development of high‐performance aqueous batteries capable of exploitation (especially in such areas as flexible wearable devices).

## 4 Experimental Section

##### Materials

Cellulose (deacetylation: 85%), acrylate (AA, 99%), acrylamide (AAm, 99%), ammonium persulfate (APS, 96%), a polytetrafluoroethylene preparation (PTFE, 60 wt% dispersion), sodium hydroxide (NaOH, 98%), zinc oxide (ZnO, 98%), zinc sulfate monohydrate (ZnSO_4_, 99%), and manganese sulfate monohydrate (MnSO_4_, 98%), graphene oxide (GO, single layer ratio: approx. 90%, diam.: 0.5–5 µm, thickness: 0.8–1.2 nm) were all purchased from J&K China Chemical, Ltd and used without further purification. The CC was purchased from Shanghai Hesen Electric Co., Ltd (China).

##### Characterizations

SEM (Hitachi S‐8010) and TEM (JEM‐2100F & JEM‐ARM300F GRAND ARM with double Cs correctors) were conducted to enable a comprehensive analysis of morphology, structure, and EDS of the samples. XPS was conducted on a PHI‐5700ESCA. XRD patterns were obtained via a Bruker Smart 1000 (Bruker AXS, Inc.). Optical images were collected using a Canon M6 (18‐150). All cycle stability and battery data in this paper were measured using LANBTS BT‐2016S and CHI‐660E.

##### Synthesis of Mn Electrode (CMOs)

The CC was cut into rectangles (1.0 × 1.0 cm^2^), which were carefully washed with 0.2 m of hydrochloric acid, then subsequently with de‐ionized water, ethanol, and acetone to remove impurities. The Mn electrode was synthesized in a conventional three‐electrode system consisting of 1.0 × 1.0 cm^2^ cleaned CC as working electrode, a platinum plate as counter electrode, and an Ag/AgCl electrode as reference electrode in MnSO_4_ aqueous solution (0.2 m). Here, high potential was used to oxidize Mn^2+^ directly onto CC to MnO_2_ and MnOOH at 1.8 V for 400 s. Then, the obtained manganese oxide was washed with water and dried at 60 °C for use. The initial mass of MnO_x_ was 5 mg, and the mass after activation was 17 mg.

##### Synthesis of Zn Electrode

The Zn electrode was synthesized using a mild electrochemical deposition in a conventional three‐electrode system consisting of 1.0 × 1.0 cm^2^ cleaned CC as working electrode, a platinum plate as counter electrode, and a Ag/AgCl electrode as reference electrode in ZnSO_4_ aqueous solution (0.2 m) at 25 °C. A deposition potential was controlled at −0.8 V for 10 min.

##### Preparation of Polyacrylamide‐Cellulose (PAMS) Hydrogel^[^ ^19^ ^]^

An amount of 0.30 g AAm was dissolved in 2 mL cellulose solution (40 mg mL^–1^), and APS (1.0 wt% of AAm) was then added into the mixture. After magnetic stirring to a clear and homogenous solution, the solution was degassed and sealed under N_2_ to remove dissolved oxygen. The free‐radical polymerization was processed at 50 °C for 12 h to form the hydrogel.

The as‐prepared hydrogel was soaked in deionized water for 12 h to remove the unreacted monomers, and then was fully dried in an oven at 50 °C for 24 h. Afterwards, the dried hydrogel was soaked in an aqueous solution (Zn:Mn = 2: 0.5) for ≈24 h before using.

##### Preparation of Sodium Polyacrylate‐ZnO (PAANa‐ZnO) Hydrogel.^[^ ^25^ ^]^

An amount of 0.30 g AA was dissolved in 2 mL 3 m NaOH solution, ZnO (0.2 m), and APS (1.0 wt% of AA) were then added into the mixture. After magnetic stirring to form a clear and homogenous solution, this was then degassed and sealed under N_2_ to remove dissolved oxygen. The free‐radical polymerization was processed in 50 °C for 12 h to form the hydrogel.

##### Density Functional Theory (DFT) Calculations

DFT as implemented in Vienna ab initio simulation package (VASP),^[^ ^26^ ^]^ was used to carry out the calculations presented here. The projector augmented wave (PAW) method^[^ ^26^ ^]^ was used to treat the effective interaction of the core electrons and nucleus with the valence electrons, while exchange and correlation were described using the Perdew−Burke−Ernzerhof (PBE) functional, plus U.^[^ ^26^ ^]^ The cut‐off energy was set at 400 eV for the plane‐wave basis restriction in all calculations. K‐points were sampled under the Monkhorst−Pack scheme for the Brillouin‐zone integration. In all calculations, the forces acting on all atoms were <0.03 eV Å^−1^ in fully relaxed structures, and self‐consistency accuracy of 10^−6^ eV was reached for electronic loops.

## Conflict of Interest

The authors declare no conflict of interest.

## Supporting information

Supporting Information

## Data Availability

The data that support the findings of this study are available from the corresponding author upon reasonable request.

## References

[1] a) D. H. Kim , N. Lu , R. Ma , Y. S. Kim , R. H. Kim , S. Wang , J. Wu , S. M. Won , H. Tao , A. Islam , K. J. Yu , T. I. Kim , R. Chowdhury , M. Ying , L. Xu , M. Li , H. J. Chung , H. Keum , M. McCormick , P. Liu , Y. W. Zhang , F. G. Omenetto , Y. Huang , T. Coleman , J. A. Rogers , Science 2011, 333, 838; 21836009 10.1126/science.1206157

[2] a) M. Song , H. Tan , D. Chao , H. J. Fan , Adv. Funct. Mater. 2018, 28, 1802564;

[3] a) J. Huang , Z. Wang , M. Hou , X. Dong , Y. Liu , Y. Wang , Y. Xia , Nat. Commun. 2018, 9, 2906; 30046036 10.1038/s41467-018-04949-4 PMC6060179

[4] a) K. Lei , Z. Zhu , Z. Yin , P. Yan , F. Li , J. Chen , Chem 2019, 5, 3220;

[5] a) A. R. Armstrong , M. Holzapfel , P. Novak , C. S. Johnson , S.‐H. Kang , M. M. Thackeray , P. G. Bruce , J. Am. Chem. Soc. 2006, 128, 8694; 16802836 10.1021/ja062027+

[6] a) W. Chen , G. Li , A. Pei , Y. Li , L. Liao , H. Wang , J. Wan , Z. Liang , G. Chen , H. Zhang , J. Wang , Y. Cui , Nat. Energy 2018, 3, 428;

[7] J. Lee , D. A. Kitchaev , D. H. Kwon , C. W. Lee , J. K. Papp , Y. S. Liu , Z. Lun , R. J. Clement , T. Shi , B. D. McCloskey , J. Guo , M. Balasubramanian , G. Ceder , Nature 2018, 556, 185. 29643482 10.1038/s41586-018-0015-4

[8] a) N. Yabuuchi , M. Takeuchi , M. Nakayama , H. Shiiba , M. Ogawa , K. Nakayama , T. Ohta , D. Endo , T. Ozaki , T. Inamasu , K. Sato , S. Komaba , Proc. Natl. Acad. Sci. USA 2015, 112, 7650; 26056288 10.1073/pnas.1504901112 PMC4485106

[9] C. Xie , T. Li , C. Deng , Y. Song , H. Zhang , X. Li , Energy Environ. Sci. 2020, 13, 135.

[10] a) C. Zhong , B. Liu , J. Ding , X. Liu , Y. Zhong , Y. Li , C. Sun , X. Han , Y. Deng , N. Zhao , W. Hu , Nat. Energy 2020, 5, 440;

[11] a) N. Jabeen , A. Hussain , Q. Xia , S. Sun , J. Zhu , H. Xia , Adv. Mater. 2017, 29, 1700804; 10.1002/adma.201700804 28639392

[12] R. Sahoo , D. T. Pham , T. H. Lee , T. H. T. Luu , J. Seok , Y. H. Lee , ACS Nano 2018, 12, 8494. 30044606 10.1021/acsnano.8b04040

[13] Z. Zhu , D. Yu , Y. Yang , C. Su , Y. Huang , Y. Dong , I. Waluyo , B. Wang , A. Hunt , X. Yao , J. Lee , W. Xue , J. Li , Nat. Energy 2019, 4, 1049.

[14] J. F. Parker , C. N. Chervin , I. R. Pala , M. Machler , M. F. Burz , J. W. Long , D. R. Rolison , Science 2017, 356, 415. 28450638 10.1126/science.aak9991

[15] T. Wei , Q. Li , G. Yang , C. Wang , Adv. Energy Mater. 2019, 9, 1901480.

[16] D. Bin , W. Huo , Y. Yuan , J. Huang , Y. Liu , Y. Zhang , F. Dong , Y. Wang , Y. Xia , Chem 2020, 6, 968.

[17] a) P. Hu , M. Yan , T. Zhu , X. Wang , X. Wei , J. Li , L. Zhou , Z. Li , L. Chen , L. Mai , ACS Appl. Mater. Interfaces 2017, 9, 42717; 29155554 10.1021/acsami.7b13110

[18] a) D. B. Loomer , T. A. Al , L. Weaver , S. Cogswell , Am. Mineral. 2007, 92, 72;

[19] L. Dai , O. Arcelus , L. Sun , H. Wang , H. Zhang , J. Carrasco , W. Zhang , J. Tang , J. Mater. Chem. A 2019, 7, 24800.

[20] F. Mo , G. Liang , Q. Meng , Z. Liu , H. Li , J. Fan , C. Zhi , Energy Environ. Sci. 2019, 12, 706.

[21] C. Zhong , Y. Deng , W. Hu , J. Qiao , L. Zhang , J. Zhang , Chem. Soc. Rev. 2015, 44, 7484. 26050756 10.1039/c5cs00303b

[22] W. Wei , X. Cui , W. Chen , D. G. Ivey , Chem. Soc. Rev. 2011, 40, 1697. 21173973 10.1039/c0cs00127a

[23] Y. Huang , Z. Li , Z. Pei , Z. Liu , H. Li , M. Zhu , J. Fan , Q. Dai , M. Zhang , L. Dai , C. Zhi , Adv. Energy Mater. 2018, 8, 1802288.

[24] H. Li , C. Han , Y. Huang , Y. Huang , M. Zhu , Z. Pei , Q. Xue , Z. Wang , Z. Liu , Z. Tang , Y. Wang , F. Kang , B. Li , C. Zhi , Energy Environ. Sci. 2018, 11, 941.

[25] L.‐X. Dai , W. Zhang , L. Sun , X.‐H. Wang , W. Jiang , Z.‐W. Zhu , H.‐B. Zhang , C.‐C. Yang , J. Tang , ChemElectroChem 2019, 6, 467.

[26] J. Qian , Q. Guo , L. Liu , B. Xu , W. Tian , J. Mater. Chem. A 2017, 5, 16786.

