# Supplementary material for: Jahn–Teller Distortion Induced Mn2+‐Rich Cathode Enables Optimal Flexible Aqueous High‐Voltage Zn‐Mn Batteries
Source: Adv Sci (Weinh). 2021 May 5;8(12):2004995. doi: 10.1002/advs.202004995 (PMC8224442; doi:10.1002/advs.202004995)
Supplement: Supplementary file 1 — Supporting Information [file ADVS-8-2004995-s001.pdf]

© 2021 Wiley-VCH GmbH

ADVANCED SCIENCE
Open Access

Supporting Information

for *Adv. Sci.*, DOI: 10.1002/advs.202004995

Jahn–Teller Distortion Induced Mn^{2+}-Rich Cathode Enables Optimal Flexible Aqueous High-Voltage Zn-Mn Batteries

Lixin Dai, Yan Wang, Lu Sun, Yi Ding, Yuanqing Yao, Lide Yao, Nicholas E. Drewett, Wei Zhang, Jun Tang,* and Weitao Zheng*

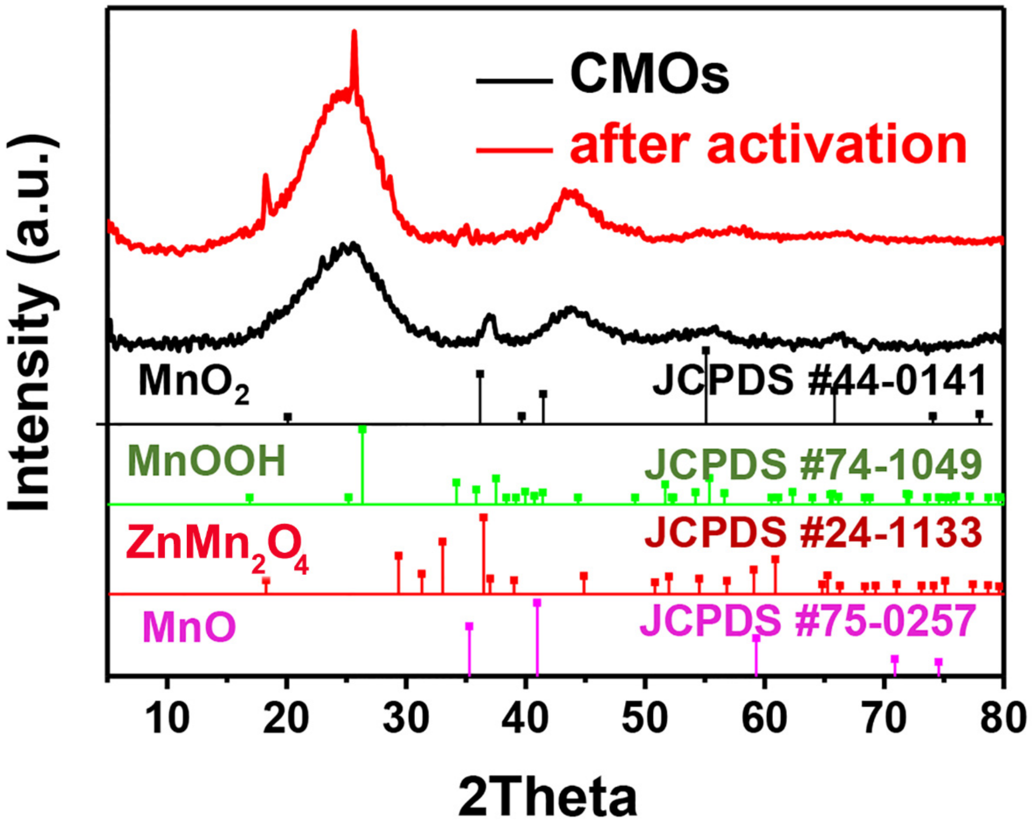

Figure S1. XRD pattern for CMOs and CMOs after activication.

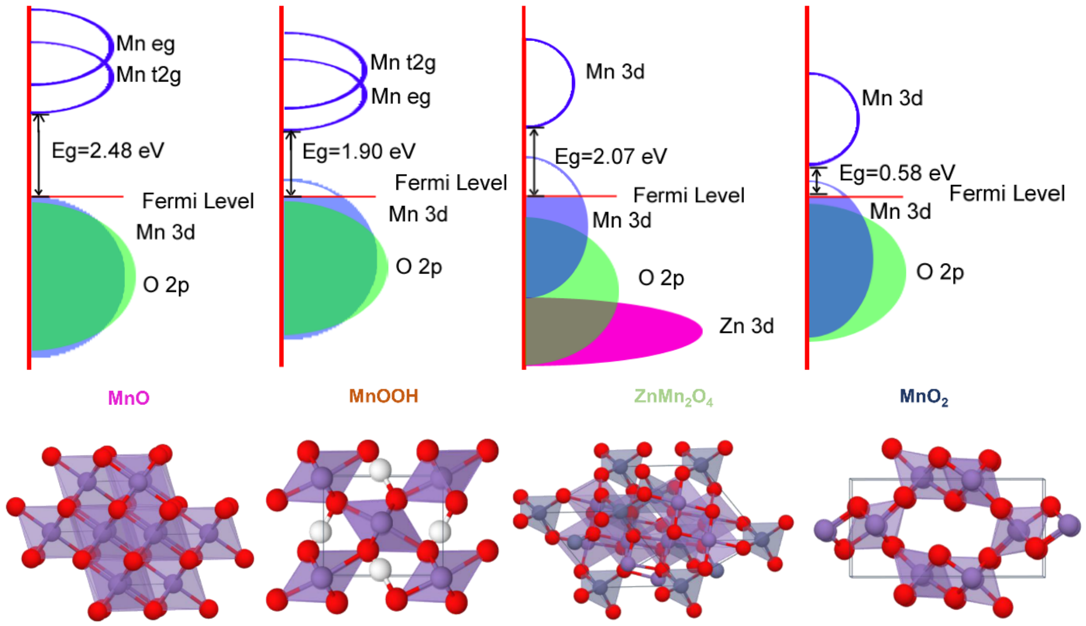

Figure S2. Schematic band structure and crystal structures of MnO, MnOOH, $ZnMn_2O_4$ and MnO_2.

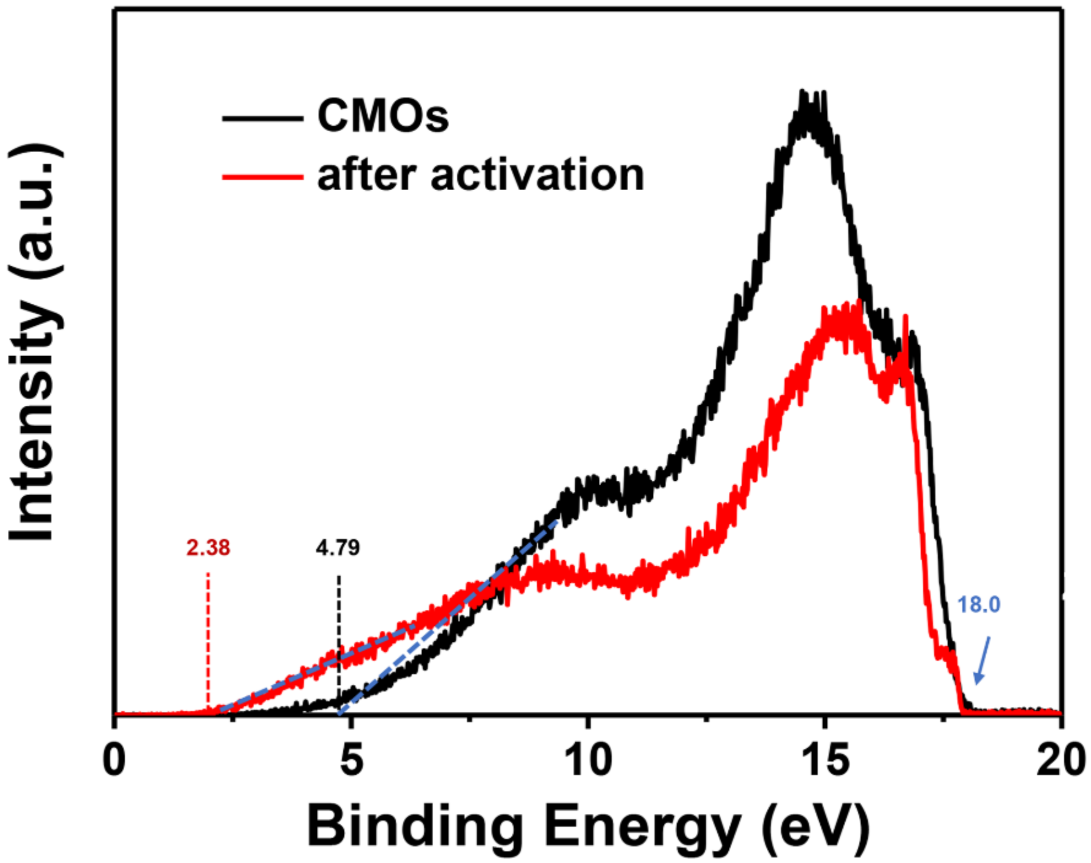

Figure S3. The Ultraviolet Photoelectron Spectroscopy (UPS) data for the original CMOs and CMOs after activation.

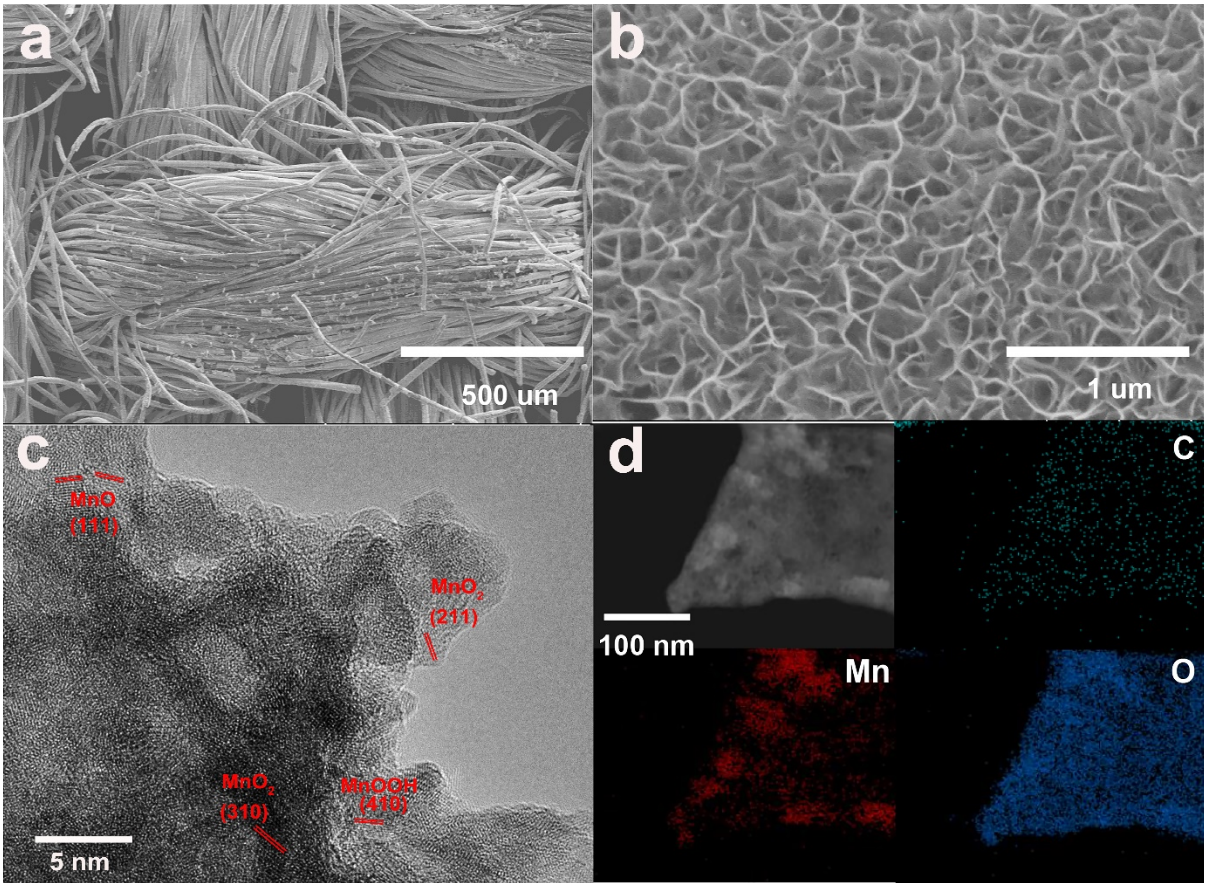

Figure S4 a) SEM image of CMOs; b) the original CMOs; c) HRTEM image of the original CMOs; d) HAADF-STEM images and corresponding C, Mn and O elemental mapping of the original CMOs.

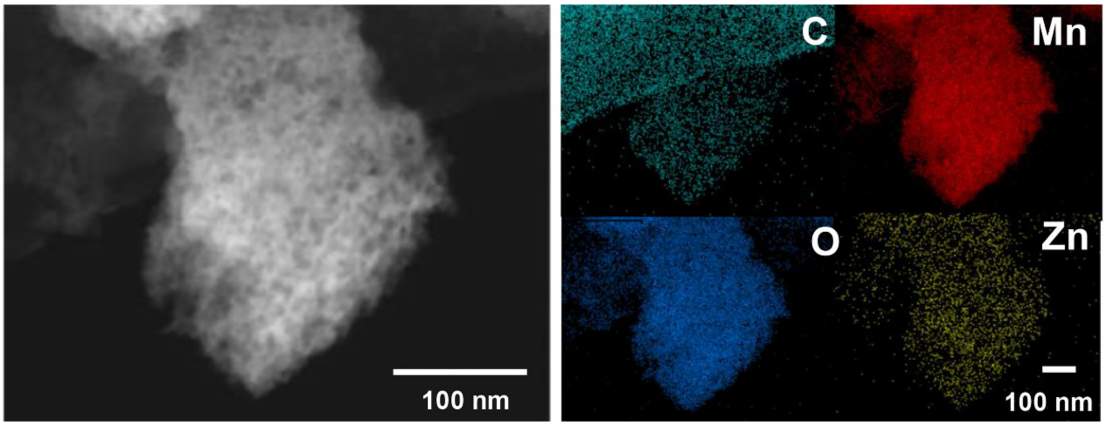

Figure S5. HAADF-STEM images and corresponding C, Mn, O and Zn elemental maps of CMOs after activation.

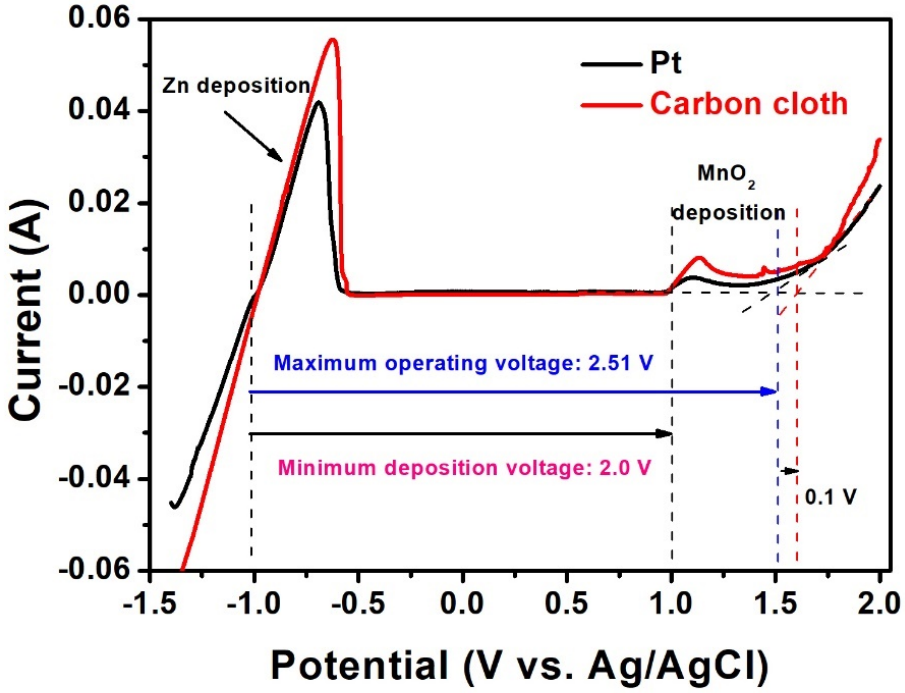

Figure S6. LSV test of 2M $ZnSO_4$ + 0.5M $MnSO_4$ to verify the potential window.

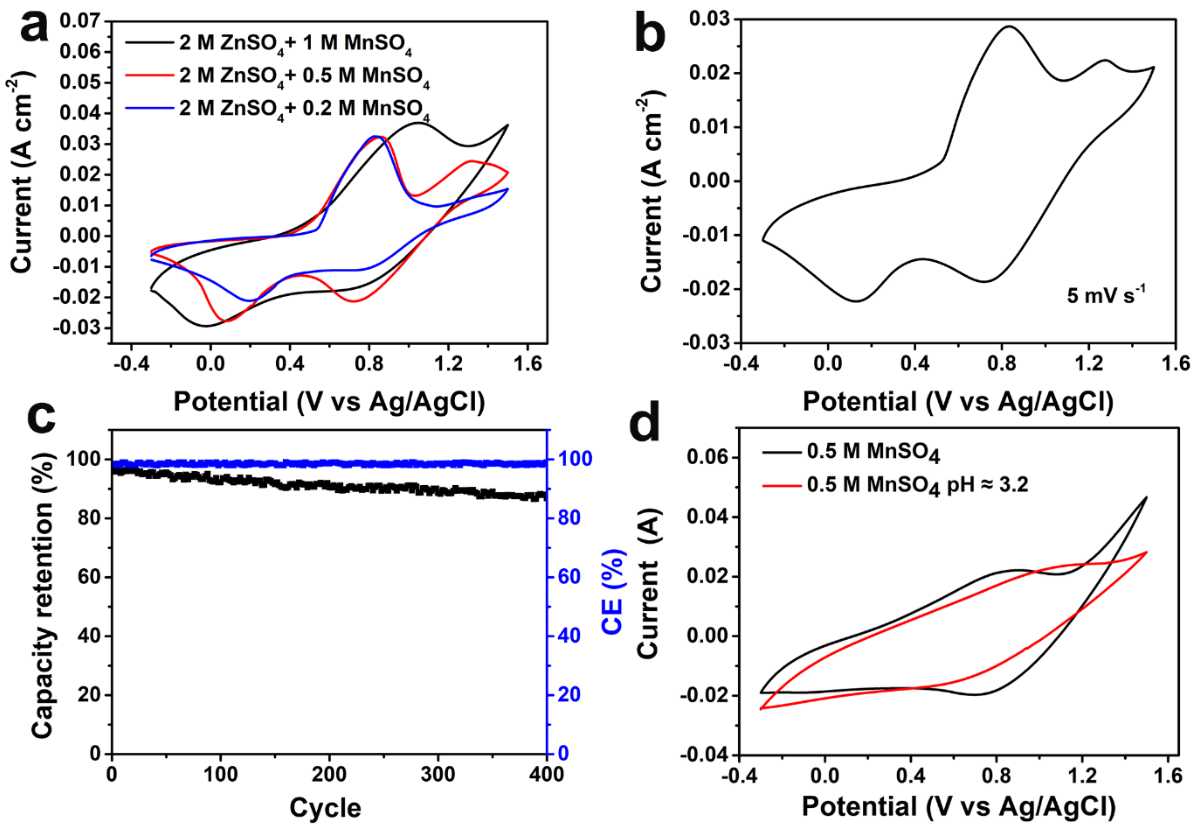

Figure S7. a) CV curves of CMOs with 2 M $ZnSO_4$ +x M $MnSO_4$ at current densities of 5 mV cm^{-2}; b) CV curves of CMOs for the first few cycles with 2 M $ZnSO_4$ at current densities of 5 mV s^{-1}; c) long-term cycling performances cycle performance at 10 mA cm^{-2}; d) CV curves of CMOs with 0.5 M $MnSO_4$ and 0.5 M $MnSO_4$ (pH≈3.2) at current densities of 5mV s^{-1}.

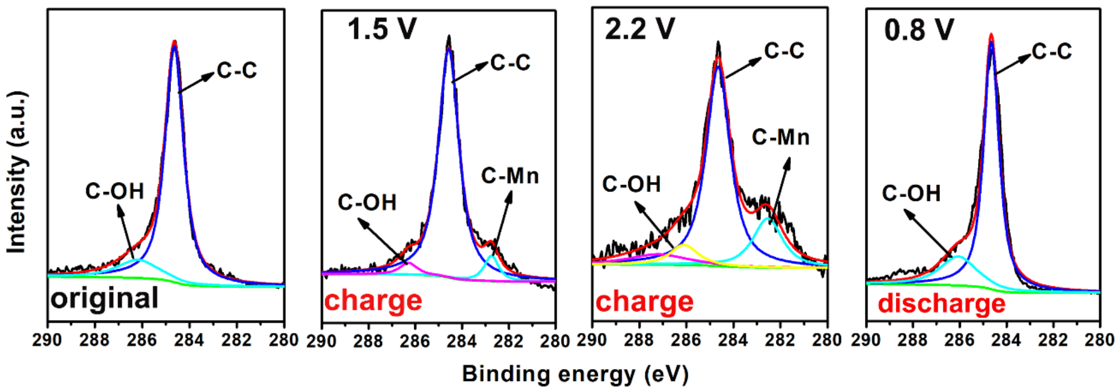

Figure S8. XPS of C 1s for CC employed as a cathode, during the charge/discharge process.

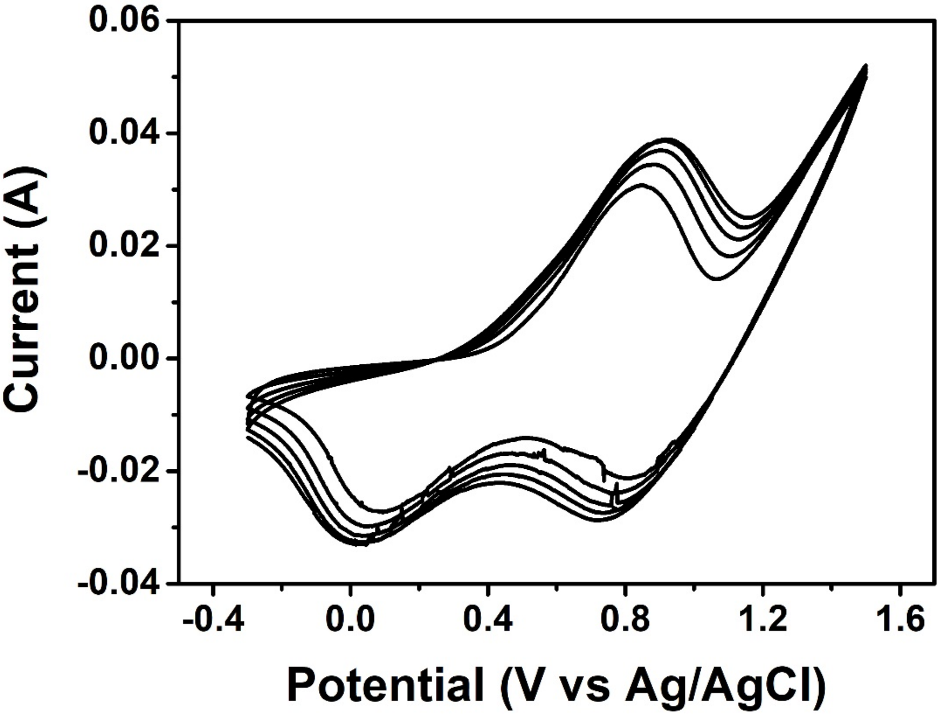

Figure S9. 10-cycle CV curves of CMOs coated with GO based on 2M $ZnSO_4$ +xM $MnSO_4$ at current densities of 5mV cm^{-2}.

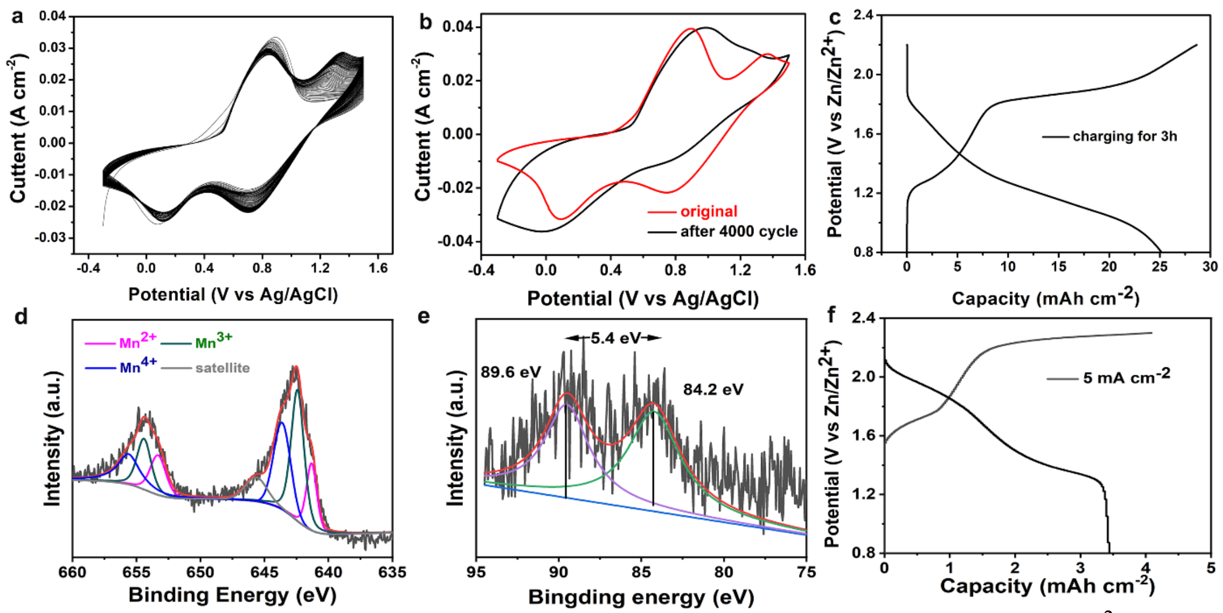

Figure S10. a) CV curves of CMOs with 2M $ZnSO_4$ + 0.5M $MnSO_4$ at of 5mV cm^{-2}; b) CV curves of original CMOs and CMOs after 4000 cycle at of 5mV cm^{-2}; c) GCD profiles for CMOs in 10 mL electrolyte. XPS of d) Mn 2p e) Mn 3s for CMOs after 4000 cycles. f) GCD profiles for CMOs with 2M $ZnSO_4$ + 0.5M $MnSO_4$ at 2.3V.

In order to increase the stability of the battery, we have reduced the electrochemical window to 2.2 V, because long-term cycling may cause severe side reactions. If the electrochemical window is too high, a rapid decline in battery performance may occur. As shown in Figure S10f, if the voltage is set to 2.3V, the coulombic efficiency is only 90.2%, and if it is cycled for a long time, the cycle stability is very poor.

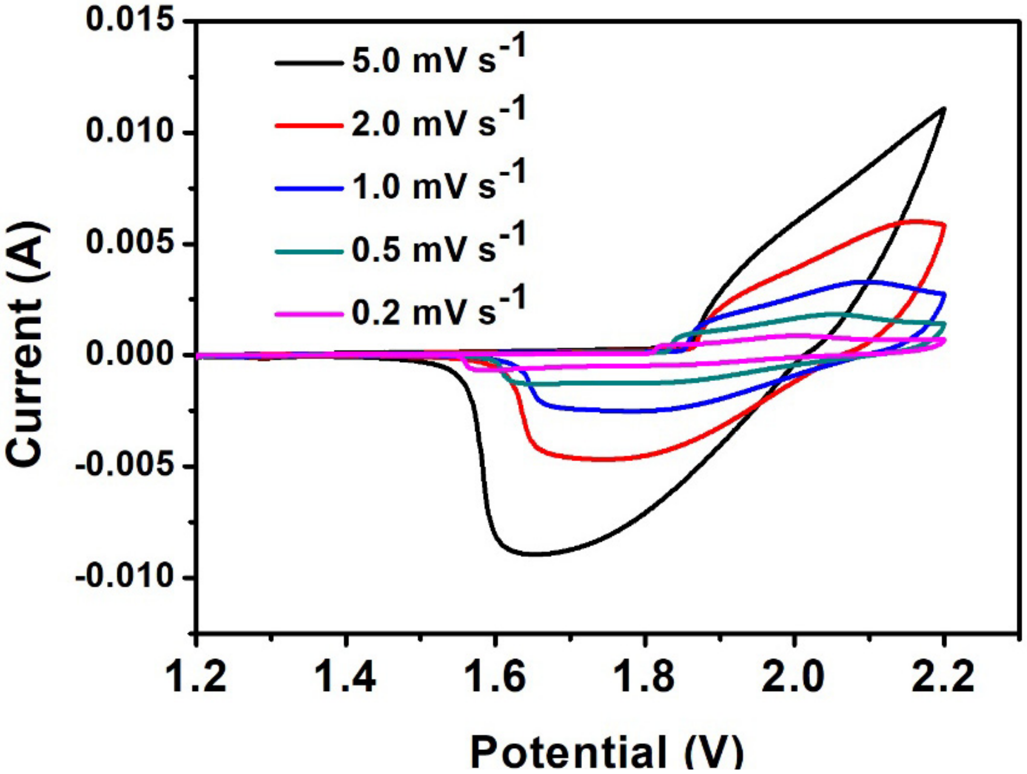

Figure S11. CV curves of ZCBC at different scan rates.

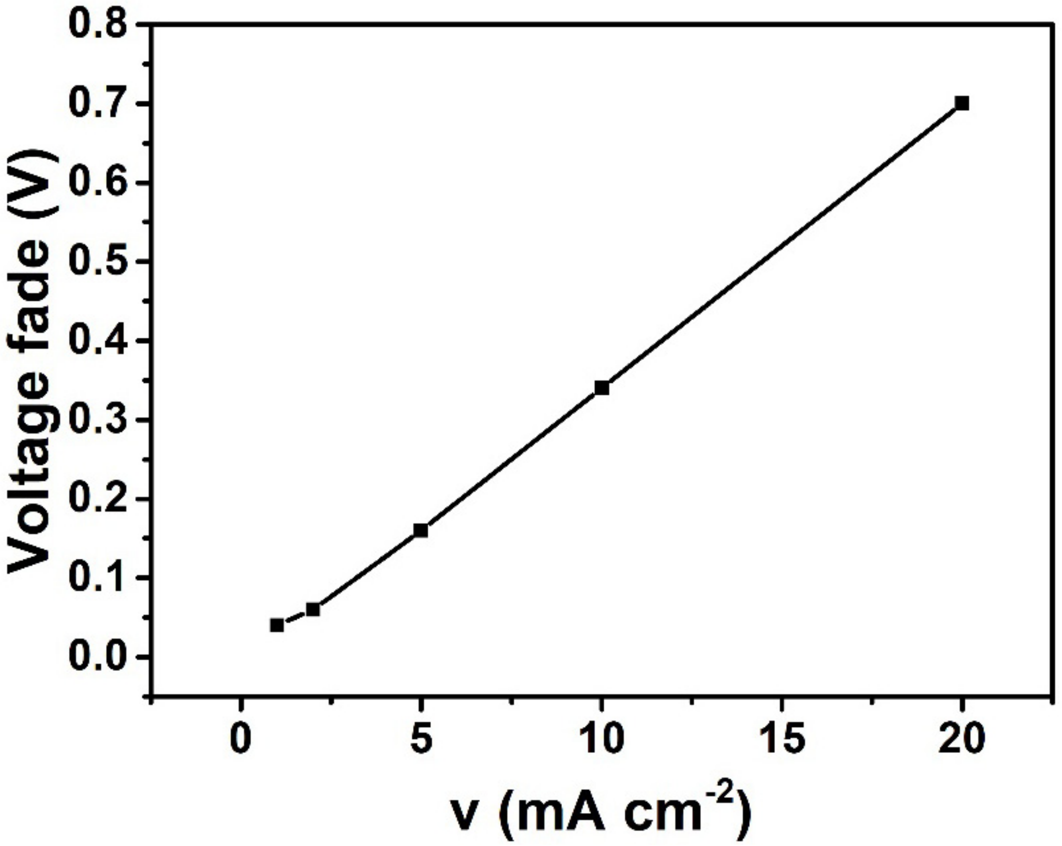

Figure S12. The voltage fade of ZCBC for GCD profiles at different charging and discharging currents.

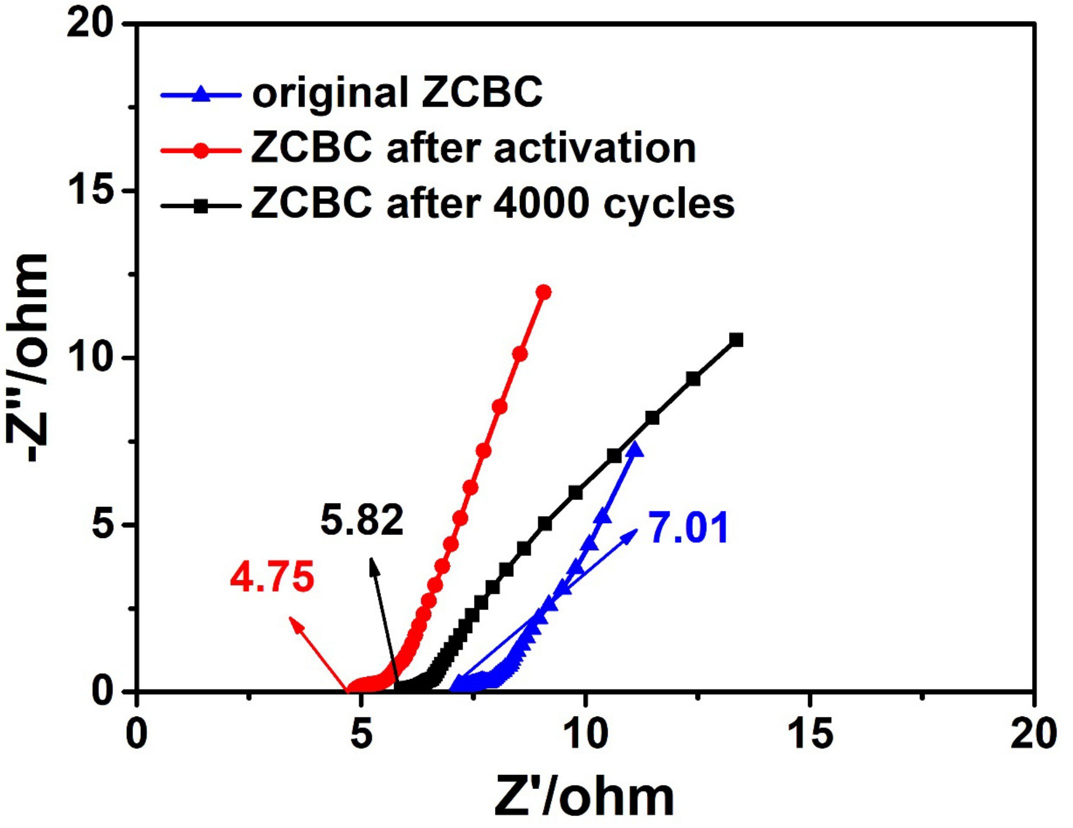

Figure S13. The EIS of original ZCBC, ZCBC after activation, and ZCBC after 4000 cycles.

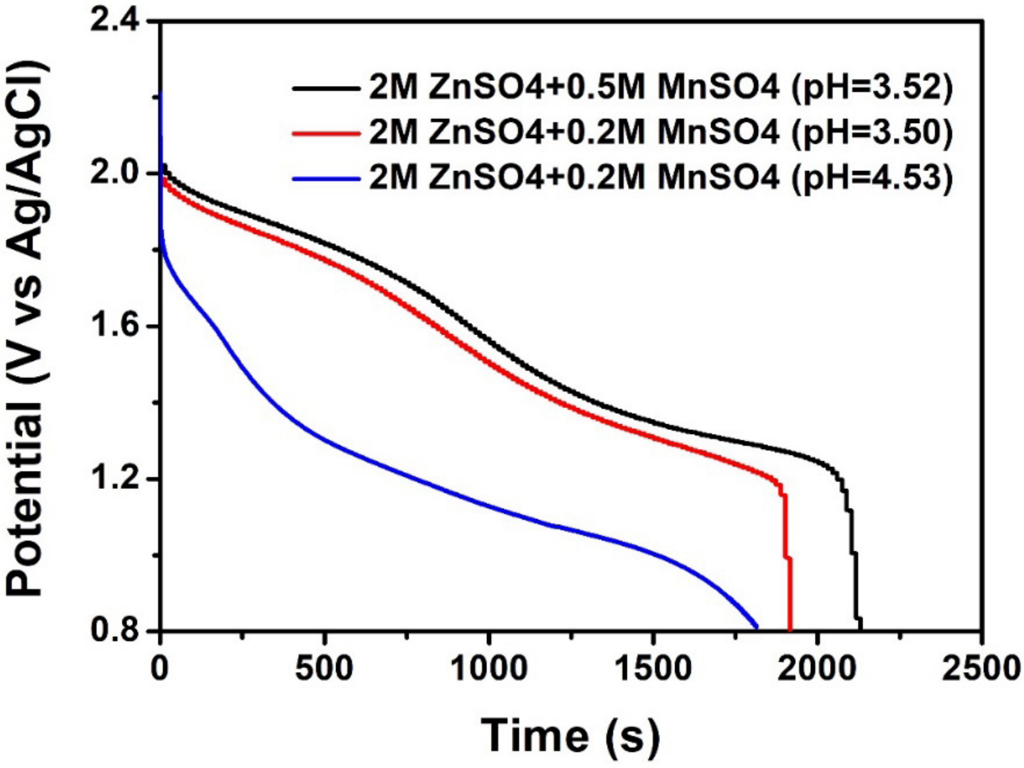

Figure S14. GCD profiles of ZCBC before and after the electrolyte replacement.

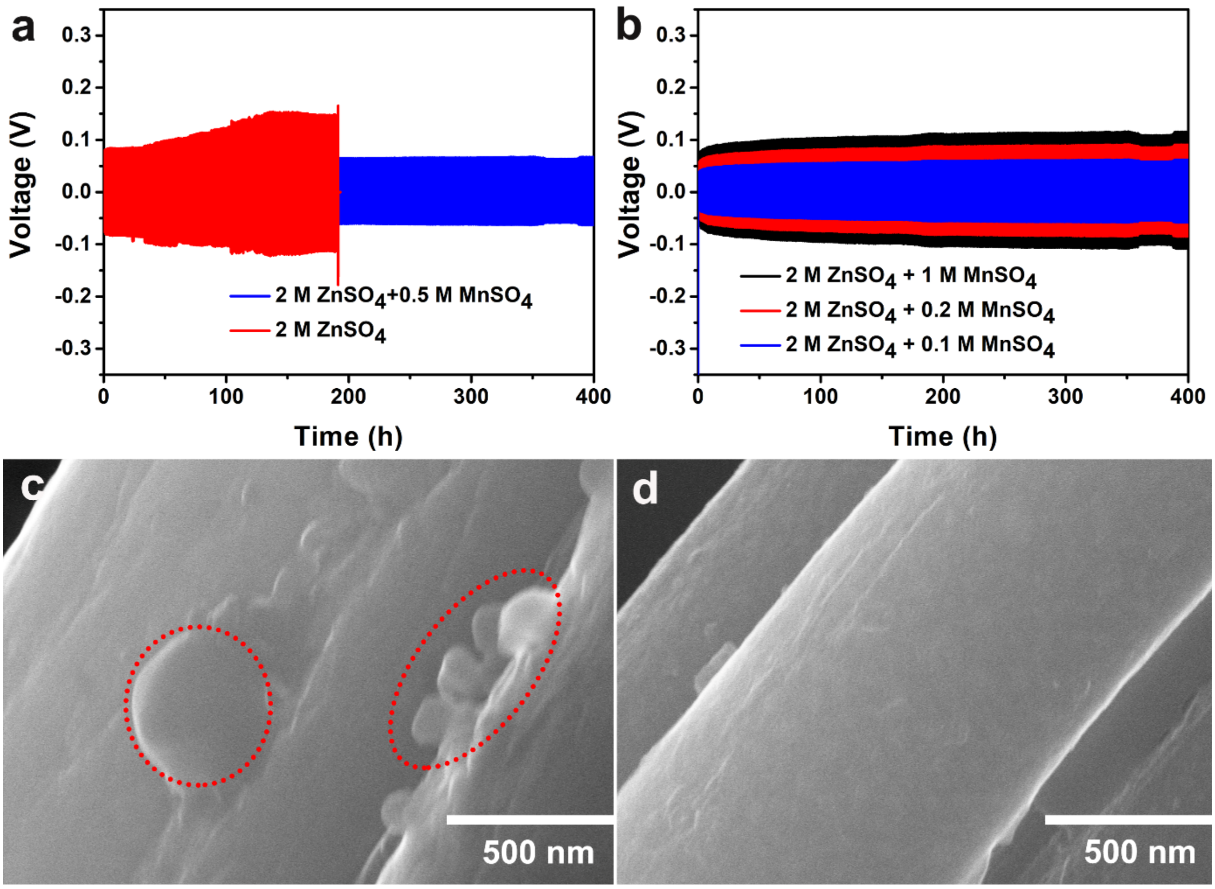

Figure S15. a) The galvanostatic cycling data for Zn/Zn cells with 2M $ZnSO_4$ + 0.5 M $MnSO_4$ (blue) and 2 M $ZnSO_4$ (red) at 0.5 mA cm^{-2}; b) the galvanostatic cycling of Zn/Zn cells with 2M $ZnSO_4$ + 1 M $MnSO_4$ (blue), 2M $ZnSO_4$ + 0.2M $MnSO_4$ (red) and 2M $ZnSO_4$ + 0.1M $MnSO_4$ (black) at 0.5 mA cm^{-2}; c) SEM image of Zn anode with 2M $ZnSO_4$ after cycling. The dendrites are marked by dashed lines; d) SEM image of Zn anode with 2M $ZnSO_4$ + 0.5M $MnSO_4$ after cycling.

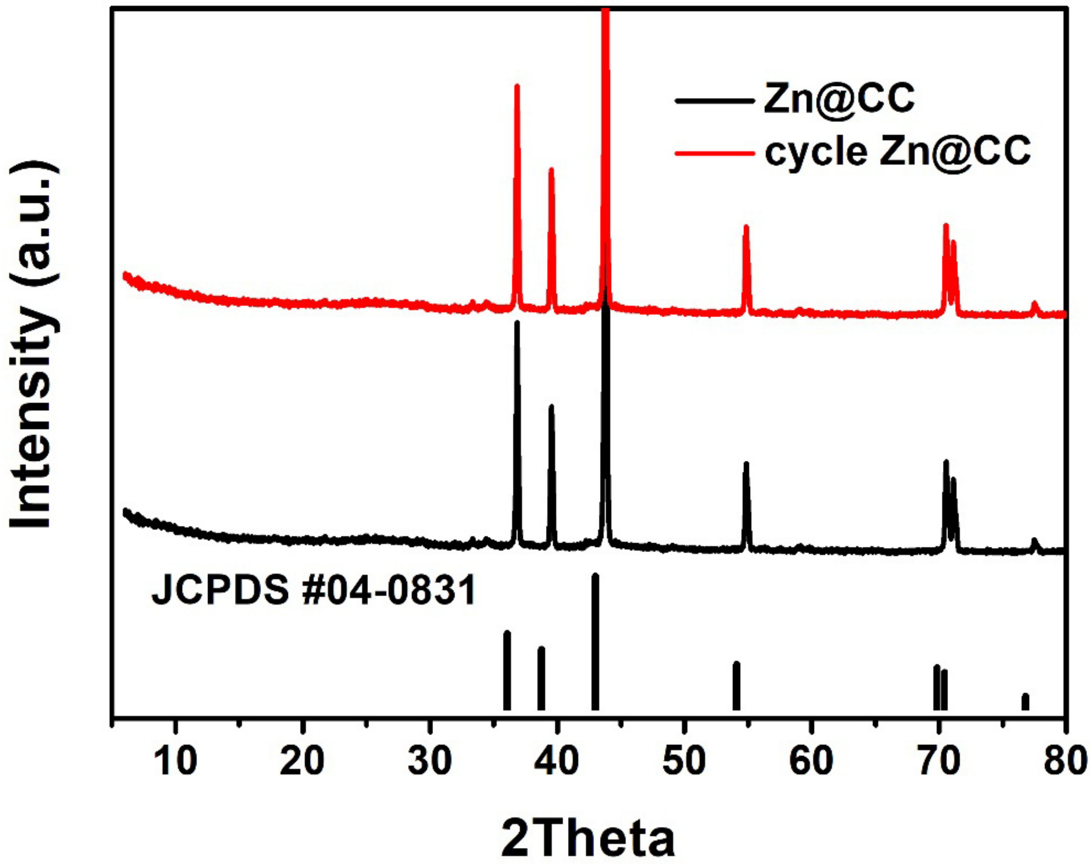

Figure S16. The XRD pattern of Zn@CC and Zn@CC after 400 h cycles with 2M $ZnSO_4$ + 0.5M $MnSO_4$.

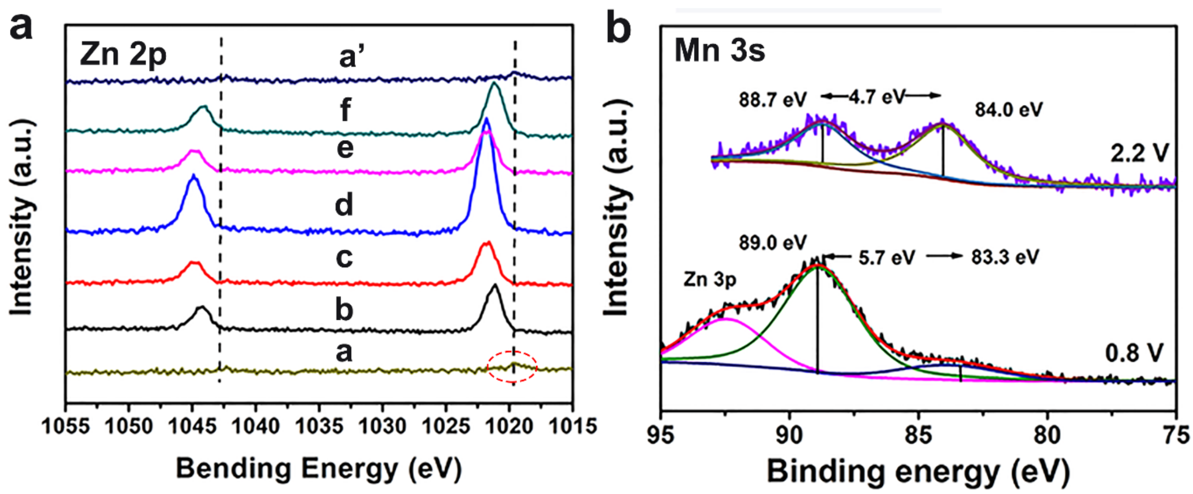

Figure S17. a) Ex-situ XPS Zn2p; b) XPS of Mn 3s for CMOs in ZCBC charging to 2.2 V and discharging to 0.8 V.

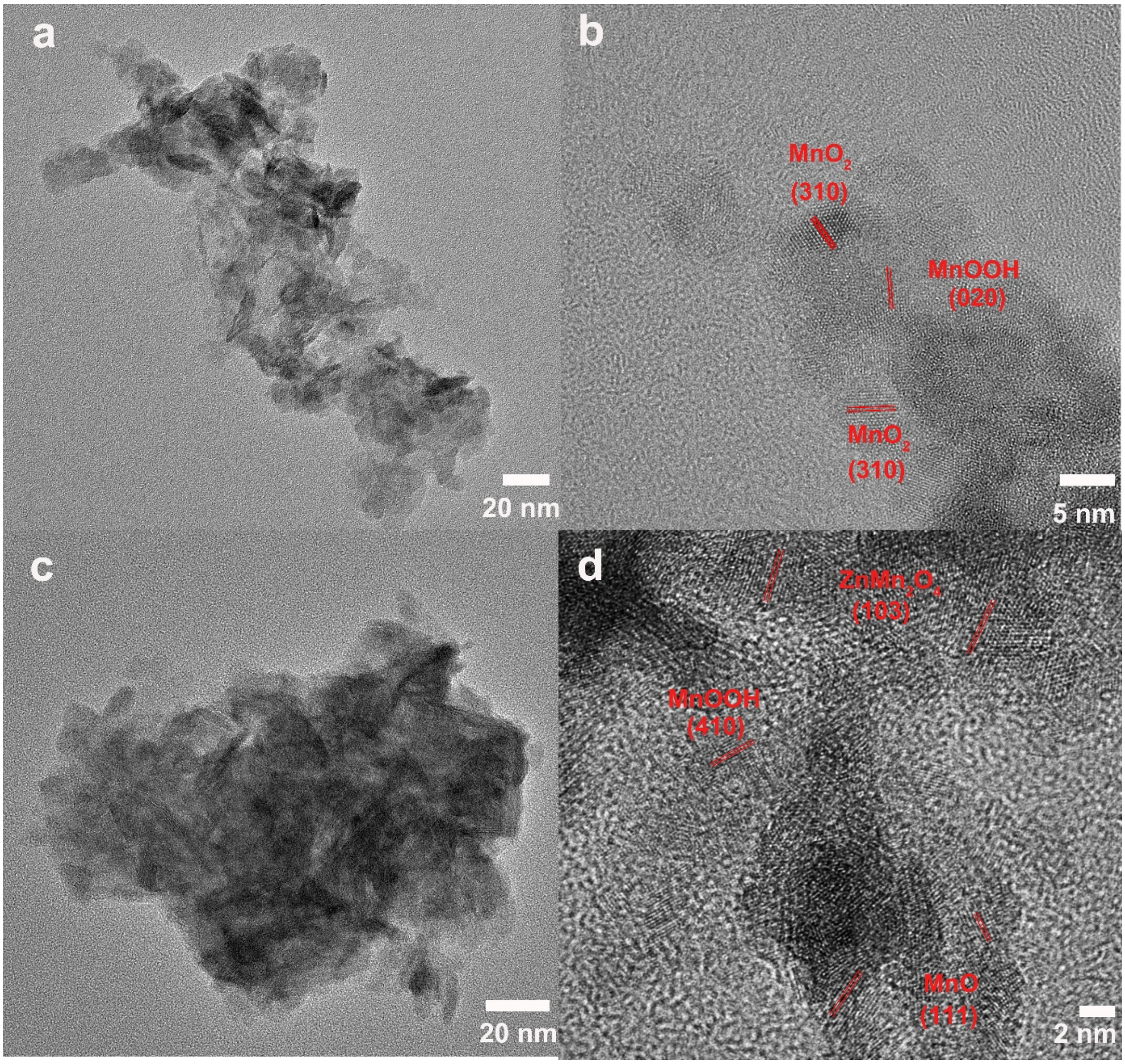

Figure S18. a), b) HRTEM images for ZCBC charging to 2.2V; c), d) HRTEM images for ZCBC discharging to 0.8V.

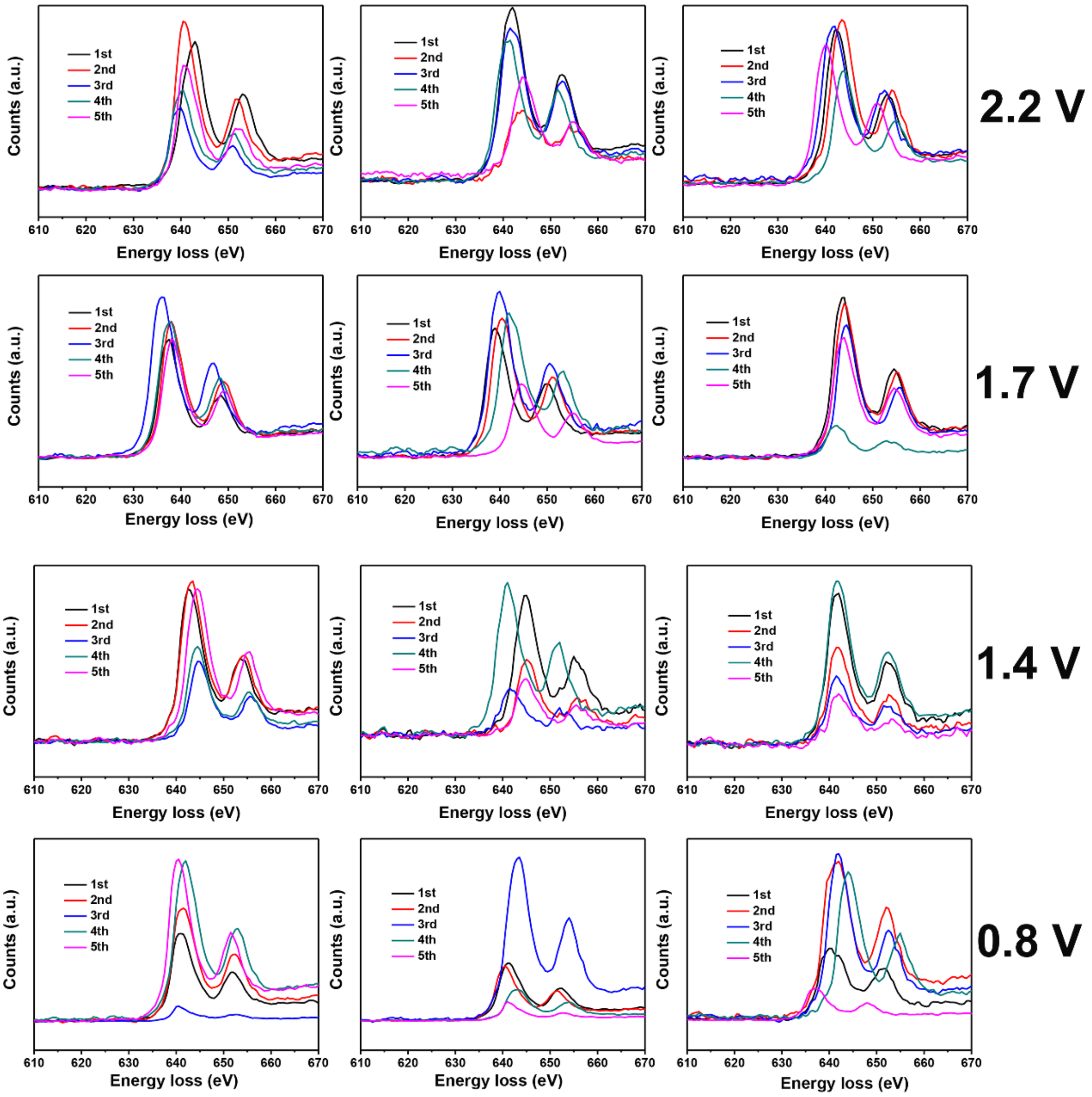

Figure S19. Mn-$L_{2,3}$ EELS spectra for ratio of Mn L_3/L_2 peak area during the discharge process.

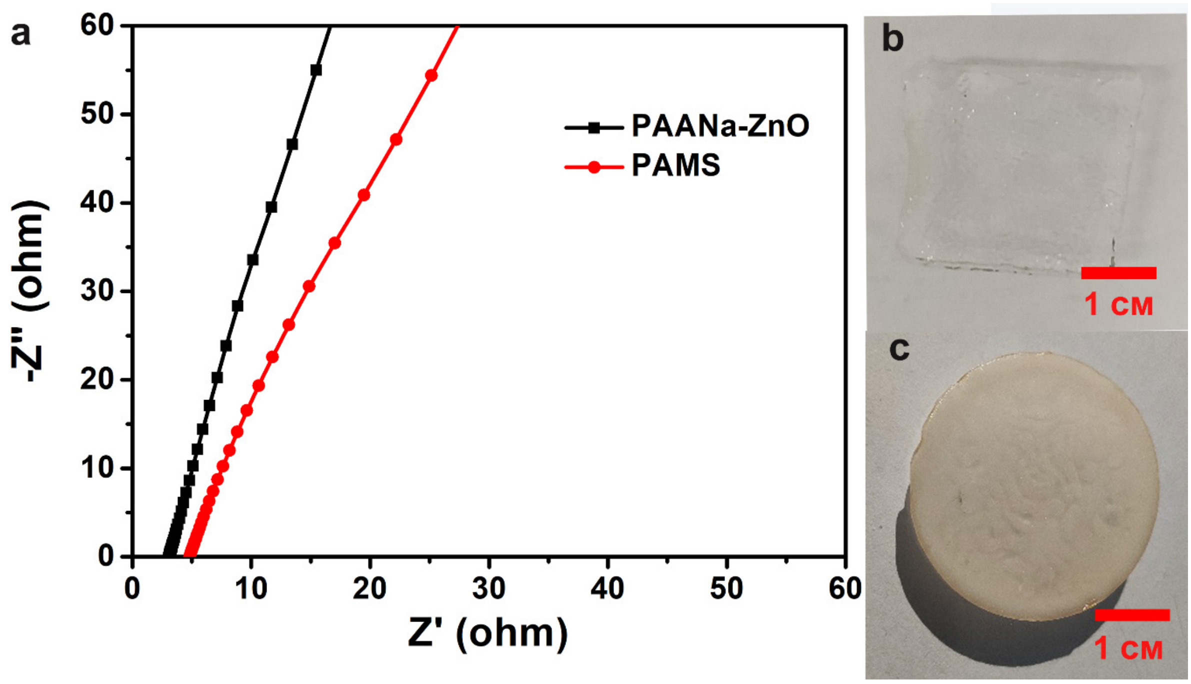

Figure S20. a) Electrochemical impedance spectroscopy for PAMS and PAANa-ZnO; digital photo for b) PAMS and c) PAANa-ZnO.

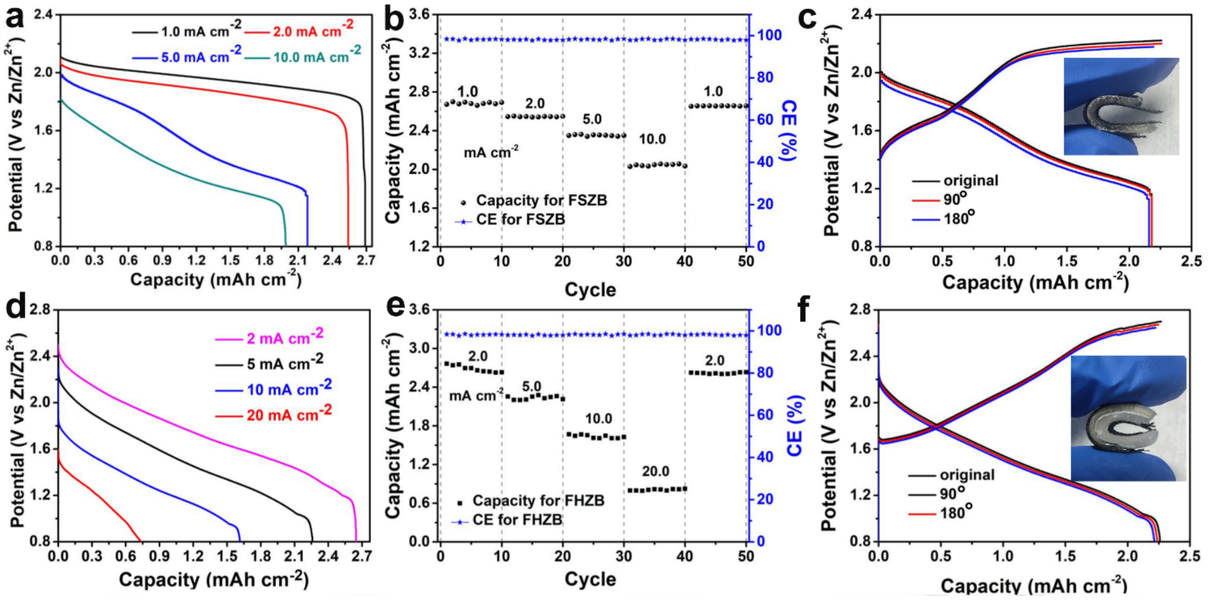

Figure S21. FSZB data of (a) GCD profiles; (b) rate performance; (c) GCD profiles of bending to different angles at 5 mA cm^{-2}; and FHZB data of (d) GCD profiles; (e) rate performance; (f) GCD profiles of bending to different angles at 5 mA cm^{-2}.

Table S1 Comparison of the electrochemical performance in different types of aqueous batteries.

Cathode/anode	electrolyte	Voltage V	Energy density mWh cm^{-2}	Power density mW cm^{-2}	Refs.
Co_3O_4@NiO//Zn	6M KOH	1.72	5.12	2.11	S1
β-$Ni(OH)_2$//Zn	6M KOH +1M LiOH + PAAS + sat. ZnO	1.80	3.25	1.66	S2
β-MnO_2//Zn	3M $Zn(OTf)_2$ + 0.1M $Mn(OTf)_2$	1.35	0.7	36	S3
NaV_3O_8//Zn	1M $ZnSO_4$ +1M Na_2SO_4	0.73	0.6	26	S4
$Na_3V_2(PO_4)_2F_3$//Zn	8M $NaClO_4$ + 0.4M $Zn(OTf)_2$	1.68	1.06	54	S5
$LiCoO_2$/Mo_6S_8	21M LiTFSI - 0.1 wt% TMSB	2.0	2.16	14.4	S6
ZCBC	2M $ZnSO_4$ + 0.5M $MnSO_4$	2.2	9.15	88	This work
FSZB	PAMS	2.2	5.74	23	This work
FHZB	PAMS/PAANa+ZnO	2.7	7.11	44	This work

References

[S1] Z. Lu, X. Wu, X. Lei, Y. Li, X. Sun, Inorg Chem Front **2015**, 2, 184.

[S2] Y. Jian, D. Wang, M. Huang, H.-L. Jia, J. Sun, X. Song, M. Guan, ACS Sus Tain Chem Eng **2017**, 5, 6827.

[S3] N. Zhang, F. Cheng, J. Liu, L. Wang, X. Long, X. Liu, F. Li, J. Chen, Nat. Commun. **2017**, 8, 405.

[S4] F. Wan, L. Zhang, X. Dai, X. Wang, Z. Niu, J. Chen, Nat Commun **2018**, 9, 1656.

[S5] W. Li, K. Wang, M. Zhou, H. Zhan, S. Cheng, K. Jiang, ACS Applied Materials & Interfaces **2018**, 10, 22059.

[S6] F. Wang, Y. Lin, L. Suo, X. Fan, T. Gao, C. Yang, F. Han, Y. Qi, K. Xu, C. Wang, Energy Environ. Sci. **2016**, 9, 3666.
